# Resistance to Inhibitors of Cholinesterase (Ric)-8A and Gα_i_ Contribute to Cytokinesis Abscission by Controlling Vacuolar Protein-Sorting (Vps)34 Activity

**DOI:** 10.1371/journal.pone.0086680

**Published:** 2014-01-21

**Authors:** Cedric Boularan, Olena Kamenyeva, Hyeseon Cho, John H. Kehrl

**Affiliations:** B-cell Molecular Immunology Section, Laboratory of Immunoregulation, National Institutes of Allergy and Infectious Diseases, National Institutes of Health, Bethesda, Maryland, United States of America; Institut de Génétique et Développement de Rennes, France

## Abstract

Resistance to inhibitors of cholinesterase (Ric)-8A is a guanine nucleotide exchange factor for Gα_i_, Gα_q_, and Gα_12/13_, which is implicated in cell signaling and as a molecular chaperone required for the initial association of nascent Gα subunits with cellular membranes. Ric-8A, Gα_i_ subunits, and their regulators are localized at the midbody prior to abscission and linked to the final stages of cell division. Here, we identify a molecular mechanism by which Ric-8A affects cytokinesis and abscission by controlling Vps34 activity. We showed that Ric-8A protein expression is post-transcriptionally controlled during the cell cycle reaching its maximum levels at mitosis. A FRET biosensor created to measure conformational changes in Ric-8A by FLIM (Fluorescence Lifetime Imaging Microscopy) revealed that Ric-8A was in a close-state during mitosis and particularly so at cytokinesis. Lowering Ric-8A expression delayed the abscission time of dividing cells, which correlated with increased intercellular bridge length and multinucleation. During cytokinesis, Ric-8A co-localized with Vps34 at the midbody along with Gα_i_ and LGN, where these proteins functioned to regulate Vps34 phosphatidylinositol 3-kinase activity.

## Introduction

In the canonical G-protein signaling, agonist binding to a G-protein coupled receptor (GPCR) triggers G alpha subunits (Gα) to exchange GDP for GTP resulting in a functional dissociation of the Gα subunit from its associated G beta-gamma (Gβγ) heterodimer [1]. This leads to the activation of downstream intracellular effector enzymes that mediate cellular responses. In non-canonical G-protein signaling, the guanine exchange factor (GEF) activity exerted by the GPCR is replaced by the action of intracellular GEFs such as Ric-8A. Ric-8A is a guanine nucleotide exchange factor for Gα_i_, Gα_q_, and Gα_12/13_ [2] and serves as a molecular chaperone required for the initial association of nascent Gα subunits with cellular membranes [3]. Ric-8A is a highly conserved cytosolic protein initially identified in *C. elegans* [4], whose functions include a regulatory role during model organism asymmetric cell division [5]–[7] and recruitment of a cortical signaling complex that helps to orient the mitotic spindle in human cells [8]. Both the canonical and non-canonical pathways utilize Regulator of G-protein Signaling (RGS) proteins to enhance the intrinsic GTPase activity of Gα_i_ [9].

In non-canonical pathways, Gα subunits are often paired with proteins containing one or more conserved GoLoco (Gα_i/o_-Loco interaction) motifs, also known as GPR or “G-protein regulatory” motifs), which acts as a Guanine Dissociation Inhibitor (GDI) much like Gβγ does in the canonical pathway [9]. A GoLoco protein Partner of Inscuteable (Pins) forms an apical protein complex essential for Drosophila neuroblast asymmetric cell division [10]. In humans, mutations in the GoLoco protein GPSM2 (also known as LGN) cause brain malformations and hearing loss in the Chudley-McCullough syndrome [11]. The non-canonical G-protein signaling proteins Ric-8A and LGN localize at the midbody during cytokinesis with Gα_i_ and RGS14 [8], [12], [13] and interference with Gα_i_ expression or with Gα_i_ nucleotide exchange prolongs cytokinesis [13]. Cytokinesis is the final step of the cell cycle leading to the physical separation of the dividing cells. Failure in cytokinesis has been shown to promote tetraploidy, aneuploidy and tumourigenesis [14].

In the present study, we identified a molecular mechanism by which non-canonical G-protein signaling proteins, particularly Ric-8A, affects cytokinesis and abscission by controlling Vps34 activity.

## Materials and Methods

### Reagents, Antibodies, Plasmids and siRNA

Hoechst 33342 (Invitrogen) was used at a concentration of 100 ng/ml, PTX (Calbiochem) was used at 200 ng/ml. MG132, Nocodazole, Roscovitine and Olomoucine were purchased from Sigma and used at 1 µM. Primary antibodies used for western blotting were the following: anti Actin-HRP conjugated (Sigma, 1∶20,000), anti GFP (Cell Signaling, 1∶1,000), anti cyclinB1 (Cell Signaling, 1∶1,000), anti phospho-p190-progesterone receptor (Invitrogen, 0.5 µg for IP), anti-ubiquitin K63 linked-HRP conjugated (Enzo, 1∶1,000); anti total ubiquitin-HRP conjugated (Santa Cruz, P4D1, 1∶1,000); anti-Gα_i3_ rabbit polyclonal (Santa Cruz, 1∶500), anti-Gβ_1_ rabbit polyclonal (Santa Cruz, 1∶50), anti-Gγ5 rabbit polyclonal (Santa Cruz, S14, 1∶100), anti-Ric-8A rabbit polyclonal (kind gift from Dr. G. Tall, University of Rochester, 1∶20,000 or Abcam, 1∶1,000 for WB or 0.5 µg for IP), anti-phospho Histone H3-Serine 10 (Cell signaling, 1∶1,000), anti-phospho serine (Cell signaling, 1∶300), anti-Vps34 (Echelon, 1∶1,000) and anti-Vps15 (Bethyl, 1 µg for IP and Abcam, 1∶1000 for WB). Secondary antibodies used for western blotting were a polyclonal anti-mouse Ig conjugated to horseradish peroxidase and a polyclonal anti-rabbit Ig similarly conjugated from Cell Signaling (1∶20,000). For Immunoprecipitation experiment True-blot^©^ horseradish peroxidase polyclonal anti-rabbit IgG or anti-mouse IgG from EBiosciences were used at a 1∶10,000 dilution. Primary antibodies used for immunofluorescence were as follows: anti-Ric-8A rabbit polyclonal (Dr. G. Tall, University of Rochester, 1∶2,000); anti-Ric-8A rabbit polyclonal (Abcam, 1∶100); anti-Vps34 rabbit polyclonal (Echelon, 1∶200) and anti-Cy3 conjugated α-tubulin antibody (Sigma, 1∶1,000). The secondary antibodies used for immunofluorescence were the following: polyclonal anti-mouse Ig Alexa 488 or Alexa 568 conjugated (Invitrogen, 1∶1,000); polyclonal anti-rabbit Ig Alexa 488 or Alexa 568 conjugated (Invitrogen, 1∶1,000).

### Plasmids and siRNA

All the siRNA knockdowns were performed in HeLa cells by following a previously described protocol [15]. Briefly, cells were seeded at day 0, Amaxa kit R-nucleofected (program O-005) with 20 nM per 25 cm^2^ of siRNA at day 1, grown for 48 h, nucleofected with the same protocol at day 3 and analyzed at day 4. RGS14 siRNA silencing was performed using three RGS14 siRNAs (AACGGGCGCAUGGUUCUGGCU; AACCGAGGAGCAGCCUGUGGC; AAGGCCUGCGAGCGCUUCCAG). For Ric-8A, GRK2 and LGN siRNA silencing, ON-TARGET plus SMARTpool L-016121-01-0005, L-004325-00-0005 and L-004092-00-0005 were respectively used (nt sequences for Ric-8A: GGGGAGAUGCUGCGGAACAUU; AGAACUUUCCAUACGAGUAUU; AGGAUGCCAUGUGCGAGAUU; CAGAGGAGUUCCACGGCCAUU based on Genbank accession number NM_021932.4; nucleotides sequence for GRK2: GGGACGUGUUCCAGAAAUU; GCUCGCAUCCCUUCUCGAA; GGAAUCAAGUUACUGGACA; GCGAUAAGUUCACACGGUU based on Genbank accession number NM_001619; nucleotides sequence for LGN: UGAAGGUUCUUUGACUUA; ACAGUGAAAUUCUUGCUAA; CUUCAGGGAUGCAGUUAUA; GAACUAACAGCACGACUUA based on Genbank accession number NM_013296).

The si*CONTROL* Non-Targeting siRNA Pool #1 was used for control siRNA transfections and Gα_i1/3_ siRNA CCGAAUGCAUGAAAGCAUG were purchased from Dharmacon.

For shRNA, hairpin primers for Ric-8A (GCTGCTATGCTGACATCTCTTTCAAGAGAAGAGATGTCAGCATAGCAGTTTTTTACGCGT) or control (GTGGACAGCTTCATCAACTATTCAAGAGATAGTTGATGAAGCTGTCCATTTTTTACGCGT) were subcloned into pSIREN-DsRed vector.

Plasmids encoding for GFP-Ric-8A, Ric-8A-Cherry, GFP-Ric-8A-Cherry, Ric-8A-CFP, RLuc-Ric-8A were subcloned from previously described pcDNA-Ric-8A vector [8]. Mutagenesis of Ric-8A was performed according Stratagene recommendations using the following primers (S501A:AGCCAATGGGGATGGCTCCCCGGGGTCATC and GATGACCCCGGGGAGCCATCCCCATTGGCT; S501D:CAGCCAATGGGGATGGATCCCCGGGGTCATCT and AGATGACCCCGGGGATCCATCCCCATTGGCTG; S88A: CACCAGCCGCCAGGCCCTGCAGGCACTA and TAGTGCCTGCAGGGCCTGGCGGCTGGTG; S155A:CTGTACCGTGAGAGGGCCTTCCCCCACGATGT and ACATCGTGGGGGAAGGCCCTCTCACGGTACAG; S155D:GCTGTACCGTGAGAGGGACTTCCCCCACGATGTC and GACATCGTGGGGGAAGTCCCTCTCACGGTACAGC).

All vectors were verified by sequencing (MWG-Eurofins). Ubiquitin-AA-Venus plasmid was a kind gift from Dr. K. Pfleger (University of Western Australia). YFP- tagged Gα_i3_ wt or Gα_i3_ Q204L plasmids were previously used [13]. Photo-activable GFP plasmid (PA-GFP) was a kind gift from Dr. J. Lippincott-Schwartz (National Institutes of Health, USA). mCherry-Tubulin plasmid was a kind gift of Dr. P. Wedegaertner (Thomas Jefferson medical school, USA). 2XFYVE-GFP was a kind gift from Dr H. Stenmark (University of Oslo, Norway), and AKT-PH CFP was a kind gift from Dr. G. Bismuth (Universite Paris Descartes, France).

### RNA Isolation and Real-time PCR

RNA was isolated using the RNeasy RNA isolation kit (Qiagen) according to the manufacturer’s instructions. cDNA was synthesized using oligo(dT) and Omniscript RT (Qiagen). Expression of Ric-8A, LGN, RGS14, GRK2, Gα_i3_ and β-actin were quantified by SybrGreen quantitative PCR using following primers:

Ric-8A F CTGAAGGCCCAGGTGCTGCC, Ric-8A R ACCCTCCCGGTCACAGGGTT, LGN F CCGCGCTGGCGTGTCATTCT, LGN R TCCTGGGGACCAGGGCAACC, GRK2 F CAAGAAAGCCAAGAACAAGCAGC, GRK2 R GCGCGGCTTGTTCTTCATCTTGG, RGS14 F TCCAAAGCCCGTGACAAATCTCC, RGS14 R TGAGTCGGTGGTGGAGTTCAAGG, Gα_i3_ F CGGCAGTGGAGCGAAGCAAGATG, Gα_i3_ R TGACTCCTTCTTCAGCACTGCCAGC, β-Actin F CGACAACGGCTCCGGCATGT and β-Actin R TGGGCCTCGTCGCCCACATA.

All results are expressed as 2^−ΔΔCt^, where ΔΔCt = (Ct_target_ − Ct_β-actin_) for treated samples − (Ct_target_ − Ct_β-actin_) for control cells. Ct stands for Cycle threshold. Data are shown as the means ± sem.

### Microscopy

HeLa cells were grown on 35-mm plates (MatTek), fixed with 4% paraformaldehyde in PBS for 20 min and permeabilized with 0.2% Triton-PBS or fixed in Methanol/Acetone for 5 min depending on the antibodies used. After blocking for 1 h in 5% BSA in PBS-0.05% Tween 20, the plates were incubated 1 h with the appropriated antibody at 4°C. Cells were imaged on a Leica DMIRBE inverted microscope equipped with a TCS SP5 confocal scanner and a 63× 1.32 NA (Numerical Aperture) objective (Leica Microsystems). Deconvolution of z-stacks was achieved with Huygens software (Scientific Volume Imaging) and 3D reconstruction of deconvoluated images with the Imaris software (Bitplane). Imaris wizard to detect spots was used to quantify 2XFYVE-GFP positive vesicles (threshold of 0.8 µm diameter and intensity 1.5 fold higher than background). Live cell imaging was performed on the same microscope but a 37°C chamber, an objective heater and a 5% CO_2_ chamber were added to the system. Cells were placed in phenol red free media the day before imaging them.

### FLIM (Fluorescence Lifetime Imaging Microscopy)

FLIM measurements were undertaken with a 63× 1.32 NA Leica oil objective lens on a multiphoton Leica TCS SP5 confocal microscope. Time-resolved detection is afforded by the addition of a non-descanned detection channel with a fast photomultiplier and SPC830 time-correlated single-photon counting electronics (Becker and Hickl GmbH, Berlin, Germany). Laser power was adjusted to give an average photon counting rates of the order 10^4^–10^5^ photons s^−1^ with peak rates approaching 10^6^ photons s^−1^, below the maximum counting rate afforded by the TCSPC electronics to avoid pulse pile-up. Acquisition times of the order of 120 s at low excitation power (1%) were used to achieve sufficient photon statistics for fitting, while avoiding significant photobleaching. Excitation was at 850 nm for EGFP lifetime measurements. Fluorescence intensity was fitted to mono-exponential decay curves on SpcImage 2.9 (Becker and Hickl GmbH, Berlin, Germany) and Fluorophore lifetime (τ) was determined.

### FRAP Microscopy

A Leica TCS SP5 confocal microscope was used with a 63× 1.32 NA objective and Leica TCS software was used to control the FRAP experiment (Leica Microsystems GmbH, Wetzlar, Germany). Electronic zoom was 20–32, confocal pinhole 1.5–2 airy units for bleaching and zoom 5 for scanning and speed was kept constant at 700 mHz. EYFP and mCherry were excited using 514 nm and 561 nm laser lines, and emitted light collected at 520–555 nm and 590–750 nm (EYFP and mCherry imaging, respectively). For FRAP experiments, laser power within this ROI was increased to 100% during a single bleaching frame, otherwise it was kept at low levels (5–10%). No Frame averaging was used. After photobleaching, the recovery is recorded for up to 5 min at 5 s intervals. The fluorescence intensity in the region of interest (ROI) over time is saved as a table using the software. From this, each data point was normalized to the fluorescence intensity before bleaching to get the relative fluorescence intensity in a region of interest. The fluorescence recovery curve is fitted by single exponential function, given by F(t) = A(1–e^−t/τ^)+B where *F(t)* is the intensity as a function of time *t*; *A* and *B* are the amplitudes of the time-dependent and time-independent terms, respectively; τ is the lifetime of the exponential term (time constant), and the recovery rate is given by *R = 1/*τ.

### Western Blotting and Immunoprecipitation

Transiently transfected HeLa cells growing in T25 flasks were lysed on ice for 1 h in 50 mM HEPES, pH 7.4, 250 mM NaCl, 2 mM EDTA, 1% NP40, 10% glycerol, containing Complete™ protease inhibitors (Roche) and supplemented with 100 µM orthovanadate sodium. After centrifugation at 13000×g for 10 min, the supernatant was collected and protein concentration determined (OD_λ = 280 nm_ measurement Nanodrop, ThermoScientific). For immunoprecipitation, supernatants (50 µg) were incubated with 1 µg anti-GFP antibody (Cell Signaling), 0.5 µg anti-p190 antibody (Invitrogen), 0.5 µg anti-Vps34 (Echelon) antibody, 0.5 µg anti-Vps15 antibody (Bethyl) or 0.5 µg anti-Ric-8A antibody (Abcam) overnight at 4°C and then incubated with protein G-Sepharose (Sigma) beads for 1 h at 4°C. After centrifugation, the immuno-bead-bound material was washed three times with lysis buffer. The immunoprecipitates were resolved on 4–20% Tris-Glycine gels. After transfer on nitrocellulose, blots were probed with the appropriate antibodies. Immunoblots were revealed by luminescence (ECL, Amersham Bioscience).

### BRET (Bioluminescence Resonance Energy Transfer) Assays

HeLa cells were transfected with 100 ng/well of the DNA construct coding for BRET donor (RLuc Ric-8A) and increasing (100–1000 ng/well) amounts of the construct coding for BRET acceptor (Ubiquitin AA- Venus or empty plasmid). DNA amount was kept constant using an empty pcDNA3.1 plasmid (Invitrogen). 24 h after transfection, cells were detached with phosphate-buffered saline/EDTA (10 mM), washed in phosphate-buffered saline and resuspended in Hanks Buffer Salt Solution. Aliquots of 10^5^ cells were distributed in 96-wells microplates (White Optiplate, PerkinElmer, for BRET measurement, Black Optiplate, PerkinElmer, for fluorescence measurement). The luciferase substrate, coelenterazine h (Promega), was added at a final concentration of 5 µM, and emitted luminescence and fluorescence was measured simultaneously using the Mithras^tm^ fluorescence-luminescence detector (Berthold, High efficiency BRET filters used 480 BP 20 nm and 540 BP 40 nm). Cells expressing BRET donors alone were used to determine background. The BRET ratio was calculated as: (emission at 540 nm/emission at 480 nm) after addition of coelenterazine h. For better readability, results were expressed in milli-BRET units (mBRET), 1 mBRET corresponding to the BRET ratio multiplied by 1000. BRET ratio were plotted as a function of (YFP/YFP_0_)/(Rluc/Rluc_0_), where YFP is the fluorescence signal at 530 nm after excitation at 480 nm, and Rluc the signal at 480 nm after addition of coelenterazine h. YFP_0_ and Rluc_0_ correspond to the same values in cells expressing the Venus fusion protein alone or the RLuc fusion protein alone respectively.

### Abscission Assay

Abscission assay was adapted from Steigemann and colleagues’ work [16]. Briefly, HeLa cells were grown in DMEM for 24 h in a Petri dish (MatTek). They were transfected using GeneJuice (EMD biochemicals) with the photo-activable GFP plasmid (PA-GFP). Cells were observed 24 h after transfection in a cell chamber maintained at 37°C, 5% CO_2_ on a Leica TCS SP5 AOBS confocal microscope with a ×63 oil lens. Focusing on metaphase cells using transmission light imaging, it was verified that imaging at 488 nm (Argon laser power at 30%) with a ×4 frames average did not induce activation of GFP on a single section or Z series before activation. Activation of GFP was induced during telophase by a single laser pulse at 405 nm using 100% of the laser diode during 50 ms focused in an approximately 5 µm^2^ area of 1 sister cell. This time allows only a sufficient photoactivation to detect a significant GFP signal. The delay between activation and recording of the first activated GFP images was 120 ms. After activation, the GFP signal was collected on single section at 15 s intervals during 8 min. This cycle of photoactivation and recording were repeated 10 times to cover the whole length of mitosis. To minimize photobleaching when recording the signals at 488 nm, only one image at 15% full power of an argon laser was used (512×512 pixels). Photomultiplier gain and offset values were kept constant for the image acquisitions. Quantification of the mean PA-GFP signals in the region of interest in the photoactivated sister cell and in a region of the same size in the non-photoactivated sister cells was established by ImageJ software (National Institutes of Health, Bethesda, MD, U.S.A., http://rsb.info.nih.gov/ij).

### Cell Synchronization

For synchronization studies, HeLa cells were grown at 80% confluence and after 4 to 6 h were blocked at the G_1_/S boundary by exposure to 2 mM thymidine (Sigma) for 14 h. The cells were then washed three times with phosphate-buffered saline solution, and normal growth medium was added. After 11 h, 2 mM thymidine was added for an additional 14 to 16 h. The cells were then washed three times with phosphate-buffered saline, and normal growth medium was added. This time point, corresponding to the G1/S transition, was designated time zero. G2/M enrichment was performed using nocodazole treatment (1 µM, 16 h).

### Flow Cytometry

At 48 h after transfection, HeLa cells were harvested and fixed with ice-cold 70% ethanol. After 30 min incubation with RNase at 37°C, cells were stained with propidium iodide (50 µg/ml) and/or phospho histone H3-ser10 FITC conjugated (EMD millipore, 1∶100) and cell cycle data were acquired with a FACS CANTO (Becton-Dickinson) and multinucleation or analyzed using FlowJo software.

### Cell Sorting

HeLa cells were harvested and stained with DRAQ5 (50 nM) for 10 min. Cells were washed and sorted according to the cell cycle data with a FACS ARIA cell sorter (Becton-Dickinson). Equal number of sorted cells was resuspended in 10 µL of lysis buffer per 10^5^ cells (50 mM HEPES, pH 7.4, 250 mM NaCl, 2 mM EDTA, 1% NP40, 10% glycerol, containing Complete™ protease inhibitors (Roche)).

### ELISA Based VPS34 Activity

Vps34 activity was assessed by ELISA according manufacturer recommendations (Echelon). Briefly, HeLa cells were lysed in 1% NP-40 lysis buffer (20 mM Tris pH = 7.5, 137 mM NaCl, 1 mM MgCl_2_, 10 mM MnCl_2_, 1 mM CaCl_2_, 100 mM NaF, 10 mM β-glycerophosphate, 100 µM Na_3_VO_4_, 10% glycerol, protease and phosphatase inhibitor cocktails). IP was performed with anti-Vps34 antibody (Echelon). Beads (associated with purified proteins) were washed in lysis buffer three times, followed by three washes in washing buffer (100 mM Tris-HCl pH = 7.5, 500 mM LiCl, 100 mM NaF, 10 mM β-glycerophosphate, 100 µM Na_3_VO_4_) and two washes in (10 mM Tris-HCl pH = 7.5, 100 mM NaCl, 1 mM EDTA, 100 mM NaF, 10 mM β-glycerophosphate, 100 µM Na_3_VO_4_). Beads were resuspended in 20 µl of reaction buffer (10 mM Tris pH = 8, 100 mM NaCl, 1 mM EDTA, 10 mM MnCl_2_ supplemented with 50 µM ATP prior to use), followed by addition 4 µl of freshly resuspended phosphatidylinositol (500 µM). Beads were incubated for 2 h min at room temperature. Reaction was terminated by quenching the kinase activity by adding 5 µL 100 mM EDTA and dilute in detection buffer (Echelon bioscience, K3004). PtdIns(3)P detection was revealed by a competitive ELISA (reaction products are added to the PtdIns(3)P-coated microplate for competitive binding to a PtdIns(3)P detector protein). The amount of PtdIns(3)P detector protein bound to the plate is determined through colorimetric detection (absorbance at λ = 450 nm). ELISA conditions were set up to give a specific and sensitive signal where values above 0.5 pmol in 100 µL were in the linear range of detection.

### Statistical Analysis

Data are expressed as a mean value ± s.e.m. All results were confirmed in at least three independent experiments. Data were analyzed using Student’s *t test or ANOVA* and *p*<0.05 (noted with *) was considered statistically significant.

## Results

### Ric-8A Protein Expression is Increased during G2/M Phase, Phosphorylated, and Ubiquitinated

**Figure 1:**
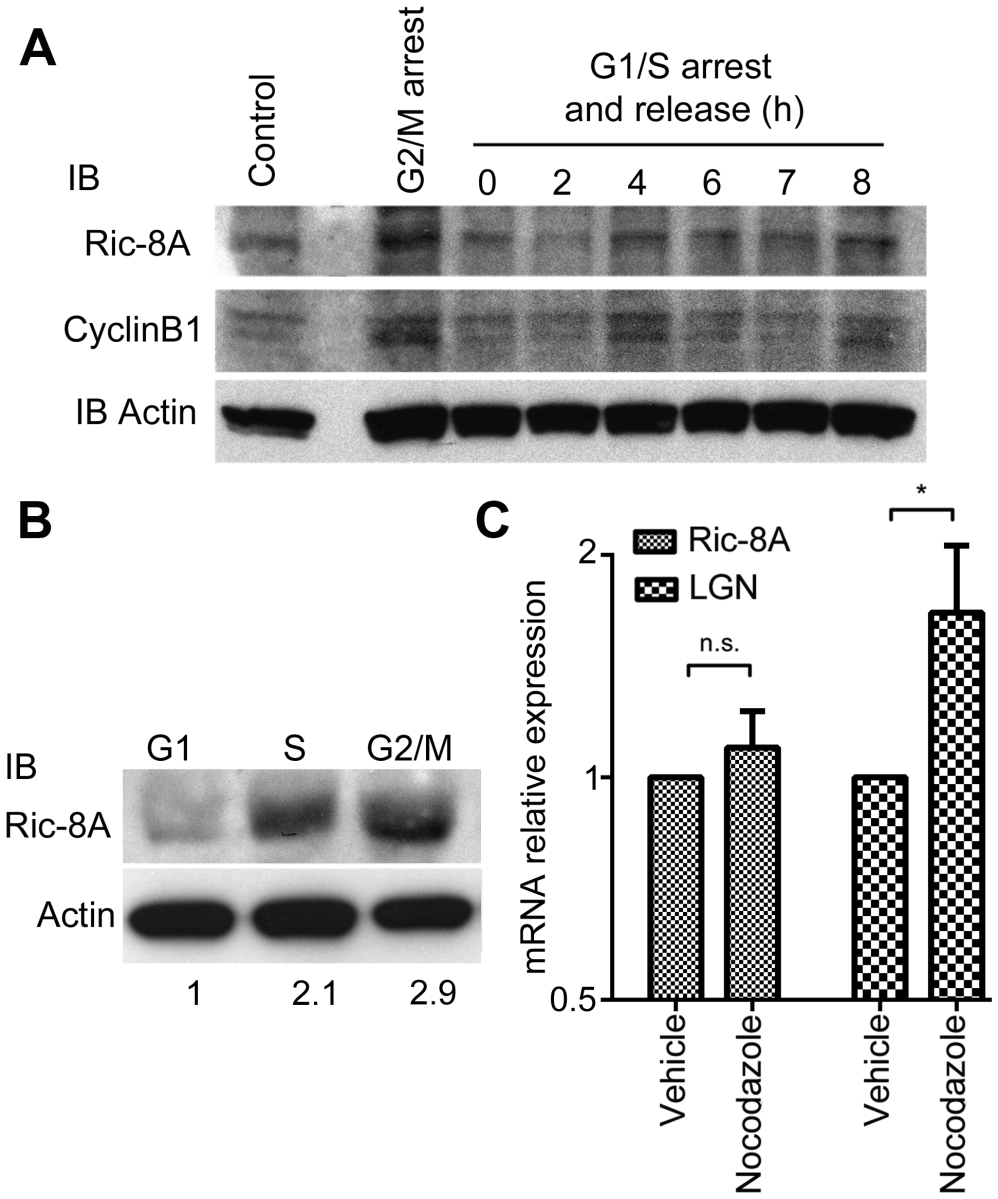
Ric-8A protein expression is increased during G2/M phase. (**A**) HeLa cells were blocked at the G1/S boundary by thymidine block and then released for the indicated times. Asynchronous or G2/M arrested cells (nocodazole 1 µM, 16 h) were used as controls. Ric-8A expression was detected by immunoblot analysis. Synchronization efficiency was verified by CyclinB1 immunoblotting. (**B**) Ric-8A and actin immunoblots of lysates prepared from HeLa cells enriched in G1, S or G2/M phase cells by cell sorting according to their DNA content. Numbers are the fold increase in Ric-8A expression normalized to actin expression using the level found in G1 phase as a baseline. (**C**) Quantification of Ric-8A and LGN mRNA expression detected by quantitative RT-PCR in nocodazole G2-M enriched HeLa cells. mRNA expression was normalized to β-actin mRNA expression, (*, p<0.05, n = 3).

**Figure 2:**
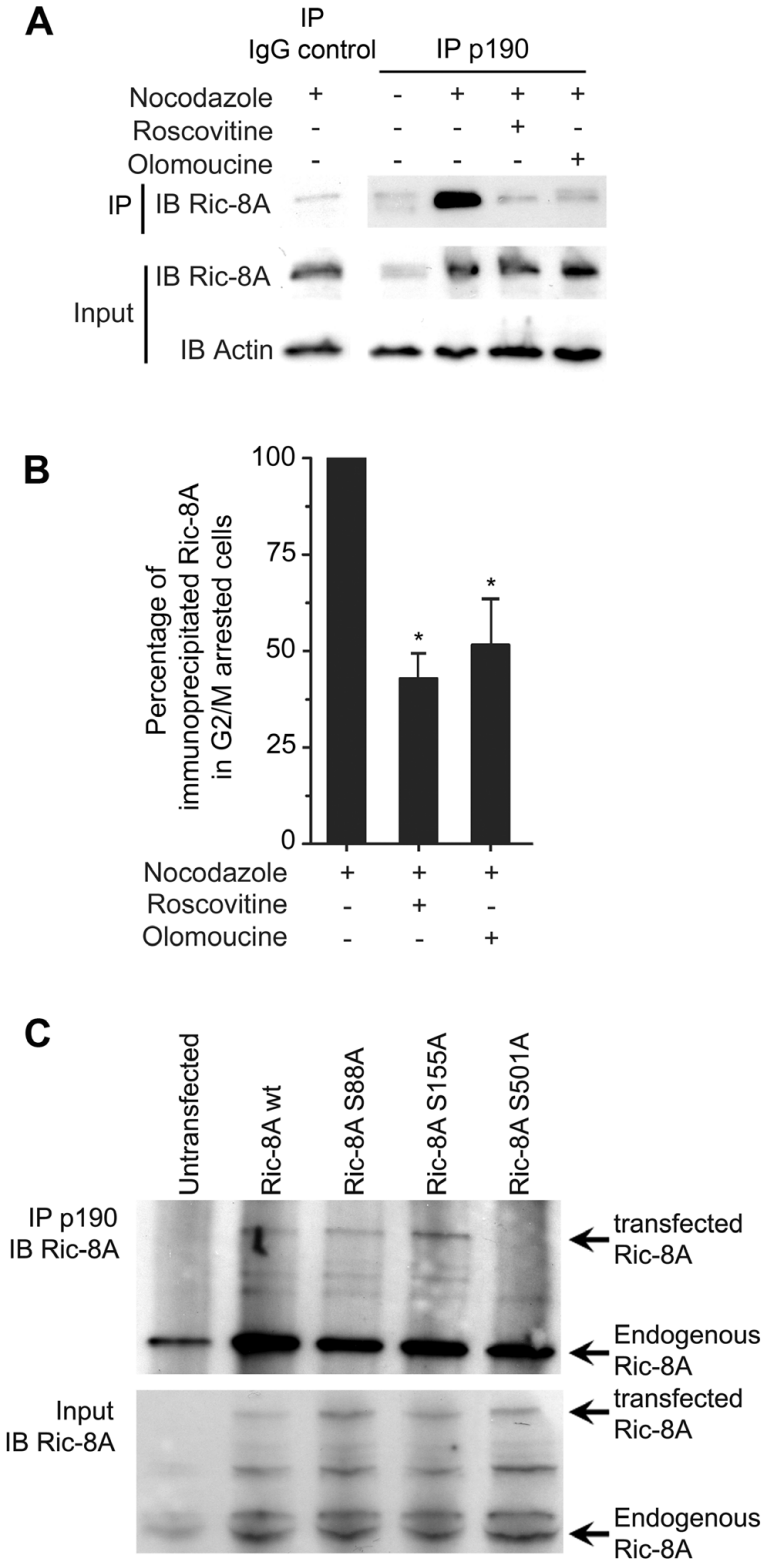
Ric-8A is phosphorylated on S501 during G2/M phase. Asynchronous or nocodazole G2/M arrested HeLa cells were treated with cyclin dependent kinase inhibitors (Roscovitine, 3 h, 1 µM or Olomoucine, 3 h, 1 µM) prior to be assessed as indicated below. (**A**) A representative Ric-8A and actin immunoblots of anti- phospho-p190 antibody immunoprecipitates prepared from HeLa cells (**B**) Graph represents the percentage of phosphorylated Ric-8A as assessed in A. Results are expressed as a percentage of the nocodazole treated condition from 4 independent experiments (*, p<0.05). (**D**) Lysates from nocodazole G2/M enriched GFP, GFP-Ric-8A wt, GFP-Ric-8A S88A, GFP-Ric-8A S155A or GFP-Ric-8A S501A expressing HeLa cells were immunoprecipitated using anti-phospho-p190 antibody, resolved on a Tris-glycine gel, and immunoblotted for Ric-8A.

To better understand the functional role of Ric-8A in cell division, we employed HeLa cells as an *in vitro* carcinoma cell line model. We first examined Ric-8A expression during different phases of the cell cycle. We found a significant variation with a peak during M phase paralleling the expression of cyclin B1 (Figure 1A) both in cells enriched in the G2/M phase following nocodazole treatment and in cells first blocked in G1/S and then released to normal growth conditions. An immunoblot analysis of Ric-8A in HeLa cells sorted for their DNA content confirmed these results (Figure 1B). During the course of these experiments, we noted that Ric-8A mRNA level remained unchanged during mitosis (Figure 1C), suggesting that Ric-8A expression was controlled post-transcriptionally. A previous study had reported that Ric-8A and several other proteins had potential consensus phosphorylation sites for CDK (S(*P*)PX[R/K/H motif), CaM kinase family phosphorylation motifs (RXXS(*P*)), or serine/proline-dependent protein kinase motifs [17]. Immunoprecipitation of cell lysates enriched for G2/M cells, or not, with a phospho-p190-PR antibody that recognizes the VLPRGLS(*P*)PARQLL phosphorylated sequence of the progesterone receptor (PR), a known substrate for CDK [18] followed by immunoblotting with a Ric-8A antibody revealed a marked enrichment of phosphorylated Ric-8A during G2/M phase. This increase was markedly reduced by roscovitine or olomoucine, inhibitors of CDK activity [19] (Figure 2A–B). In order to verify that Ric-8A undergoes serine phosphorylation, we performed the immunoprecipitation in the presence or absence of okadaic acid, a serine phosphatase inhibitor or by blotting Ric-8A immunoprecipitates with a phospho serine antibody. In both case, nocodazole G2/M enriched Hela cells showed an increase in Ric-8A serine phosphorylation (Figure S1 in file S1). The substitution of the serine at position 501 of Ric-8A with alanine revealed that this amino acid residue is involved in the epitope recognized by the phospho-p190-PR antibody (Figure 2C).

**Figure 3:**
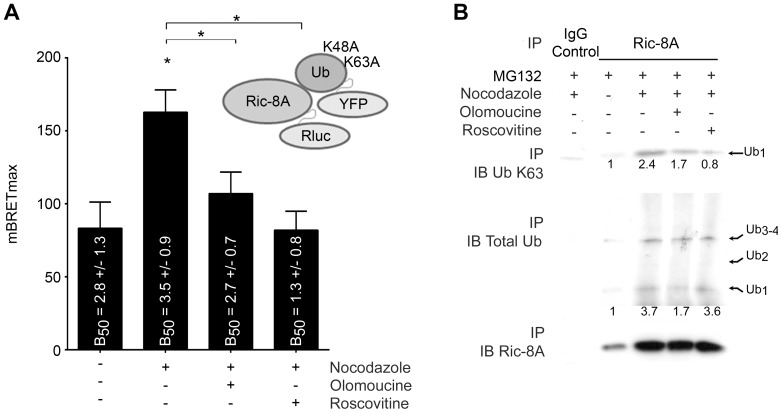
CDK activity inhibition blocks Ric-8A ubiquitinitation during G2/M phase. (**A**) HeLa cells were transiently transfected with a fixed quantity of RLuc-tagged Ric-8A and an increasing amount of Venus-Ubiquitin K63A K48A to measure if Ric-8A could be in close vicinity of mono-ubiquitin as outlined by the upper right scheme. The mBRET_max_ and the mBRET_50_ (B_50_) of the fitting curves parameters are represented on the histograms. 3 independent experiments of at least 12 donor/acceptor ratio transfections were performed (*, p<0.05). (**B**) Ric-8A immunoprecipitates of lysates prepared from MG132 (1 h, 10 µM) treated HeLa cells were immunoblotted for K63-linked ubiquitin, total ubiquitin, or Ric-8A antibodies. Nocodazole (16 h, 1 µM) and cyclin dependent kinase inhibitors (Roscovitine, 3 h, 1 µM or Olomoucine, 3 h, 1 µM) treatments are indicated. Numbers are the fold increase in Ric-8A ubiquitination using the levels found in non-treated cells as a baseline. The results were normalized to the relative amount of immunoprecipitated Ric-8A.

Since many cell cycle proteins like cyclin E and D1 are phosphorylation-ubiquitin regulated [20], [21] we checked whether Ric-8A was similarly controlled. To determine whether Ric-8A phosphorylation might alter its ubiquitination status, we measured Ric-8A mono-ubiquitination in live cells by bioluminescence resonance energy transfer (BRET) assay using luciferase tagged Ric-8A and Venus tagged K48A and K63A ubiquitin (the K48A and K63A mutations allow BRET assay and prevent formation of long ubiquitin chains [22], [23]). In G2/M enriched cells, Ric-8A is more ubiquitinated (BRET_max_ nocodazole = 162.7±15.6 vs BRET_max_ asynchronous = 71.6±10.3). Furthermore, inhibition of CDK activity decreased Ric-8A ubiquitination (BRET_max_ nocodazole+roscovitine = 88.3±13.2 and BRET_max_ nocodazole+olomoucine = 118.4±14.3) in nocodazole G2/M arrested HeLa cells (Figure 3A). We confirmed these observations by co-immunoprecipitation experiments, which showed that both K63-linked and general ubiquitination are decreased in presence of CDK kinase inhibitors (Figure 3B). These data indicate Ric-8A is phosphorylated in a cell cycle dependent fashion that likely shapes its post transcriptional regulation during G2/M phase of the cell cycle. The phosphorylation and ubiquitination of Ric-8A during G2/M may lead to its proteasomal destruction and the re-setting of Ric-8A levels for a new G1 phase.

We then examined Ric-8A expression in various cancers compared to normal tissues using a tumor array in which tissue lysates from cancer biopsies or from corresponding non-tumor tissues (age- and sex- matched) were spotted. In 8 out of 14 samples Ric-8A was under-represented in the tumor samples (testis, thyroid, rectum, liver, laryngopharynx, pancreas, stomach and skin Figure S2A–S2C in file S1) compared to normal tissues, whereas it was over-represented in the remaining cancer tissues (uterus, prostate, breast, lung, ovary and cervix, (Figure S2B–C in file S1). Even in the absence of consistent trends in various transformed tissues, these data suggest that altered Ric-8A protein expression is a frequent event during cancer development.

### Ric-8A Undergoes a Conformational Change during Mitosis

**Figure 4:**
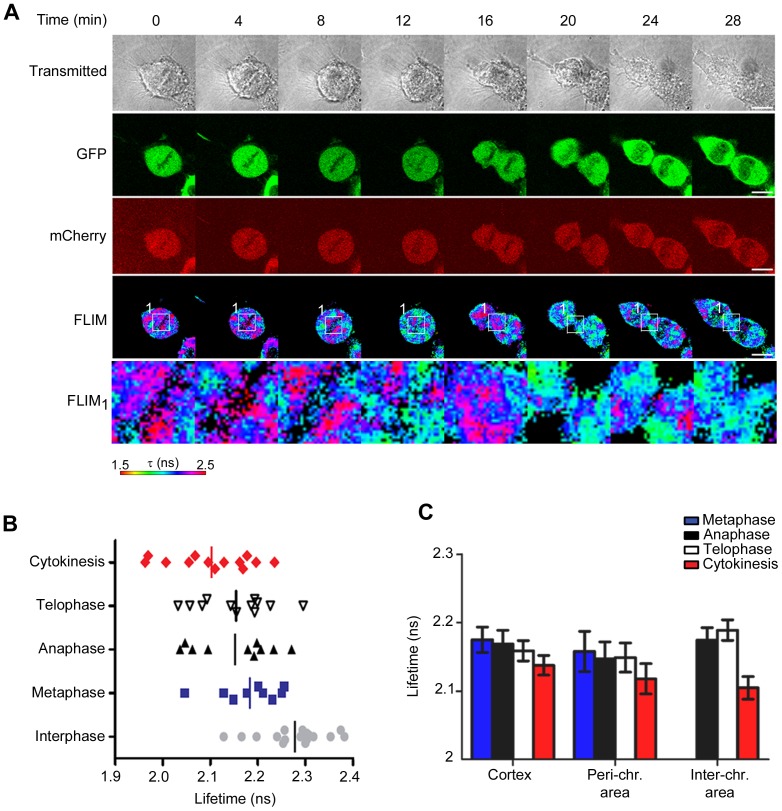
Ric-8A conformational change occurs during mitosis. (**A**) HeLa cells were transiently transfected with GFP-Ric-8A-mCherry. Focusing on metaphase cells using transmission light imaging, FRET by FLIM measurements were done on live cells. The panel shows a representative lifetime imaging and an electronic magnification of the interchromosome area (square 1) containing the midbody is displayed (**B**) Results of the average lifetime in whole cells at different stages of the cell cycle. Each point represents a single cell. (**C**) Results of the average lifetime in the different sub-cellular compartments at different stages of the cell cycle. The data were acquired from 4 independent transfections and from 6–15 cells for each bar.

A previous study indicated that during mitosis, and parallel with an increase in its expression, LGN undergoes a conformational change that alters its interaction with Gα_i_ subunits [24]. To determine if Ric-8A also undergoes a cell cycle dependent conformational change we constructed a biosensor by fusing the green fluorescent protein GFP to the N-terminus of Ric-8A and the red fluorescent protein mCherry to the C-terminus. We monitored Ric-8A intra-molecular conformational changes in living cells by fluorescence lifetime imaging (FLIM). Förster Fluorescence Resonance Energy Transfer (FRET) between a FRET-donor (GFP in the present case) and a FRET-acceptor (mCherry) accelerates the fluorescence lifetime of the light-excited FRET-donor. The reduction of fluorescence lifetime increases with FRET efficiency. First, we verified that no detectable FRET occurred via inter-molecular transfer between two adjacent Ric-8A molecules and that the observed FRET depended upon the position of GFP and mCherry in the GFP-Ric-8A-mCherry biosensor (Figure S3A–B in file S1). HeLa cells expressing the biosensor were then examined during different cell cycle phases. Compared to interphase cells the lifetime of GFP progressively decreased during mitosis reaching a minimal value during the late-telophase/cytokinesis stage (from 2.28±0.06 ns to 2.11±0.09 ns) (Figure **4**A–B). At a sub-cellular level, we examined the 3 major areas in which Ric-8A localizes in mitotic cells; the cell cortex, centromeres, and the inter-chromosomal area that contains the midbody [8]. The highest change of fluorescence lifetime was observed in the inter-chromosomal area suggesting that a major conformational change in Ric-8A occurred upon its localization to the midbody (Figure 4A–4C).

### Ric-8A Inhibition Increases the Length of the Intercellular Bridge, Delays Abscission Time and Promotes Multinucleation

**Figure 5:**
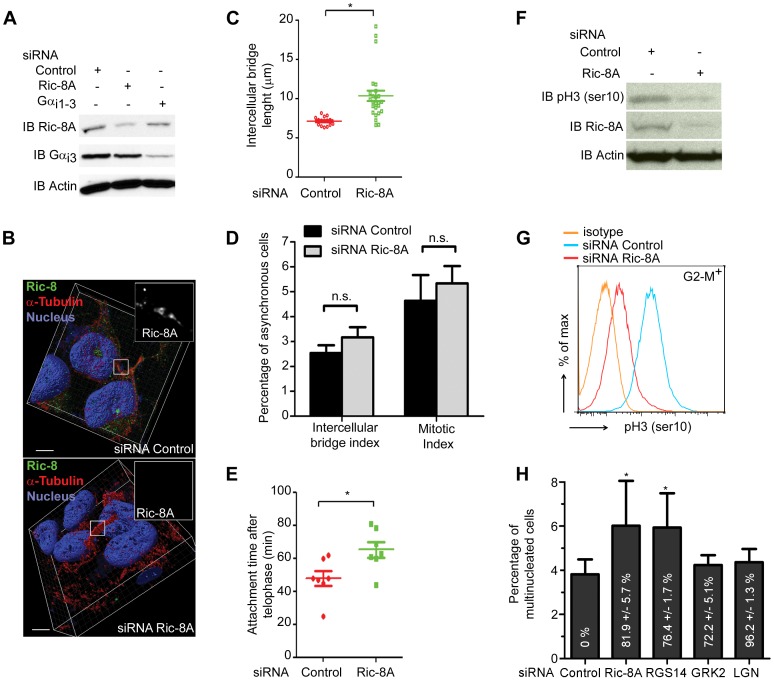
Ric-8A inhibition increases the length of the intercellular bridge, delays abscission time and promotes multinucleation. (**A**) Immunoblot of Ric-8A and Gα_i3_ protein expressions after treatment of HeLa cells with siRNA control or siRNA directed at Ric-8A or at Gα_i_ subunit 1–3. (**B**) Panels show a representative deconvolution of z-stacks and 3D reconstruction of siRNA control (top panel) or Ric-8A siRNA (bottom panel) treated HeLa cells. Scale bar is 5 µm. Magnification of midbody region and Ric-8A localization is shown in the white squares. (**C**) Intercellular bridge length quantified from 3 independent experiments (*, p<0.05) by immunostaining with an anti-α-tubulin antibody. (**D**) Distribution and the average abscission time of HeLa cells transiently transfected with siRNA-Ric-8A or siRNA-control and the photo-activable GFP plasmid. Results from 12 cells were acquired in 4 independent experiments (*, p<0.05). (**E**) Histogram represents the percentage of total of cells with a mitotic phenotype (prophase to cytokinesis) and the number of cells showing an intercellular bridge. At least 500 cells were analyzed in 3 independent experiments, (n.s. stands for non-significant). (**F–G**) HeLa cells transiently transfected with siRNA-Ric-8A or siRNA-control were analyzed for their phospho histone H3-ser10 levels either by western blotting technique (**F**) or by flow cytometry technique (**G**) focusing on G2-M positive cells. (**H**) The percentage of multinucleated HeLa cells among cells transiently transfected with siRNA Control, siRNA Ric-8A, siRNA RGS14, siRNA GRK2 or siRNA LGN was assessed by flow cytometry 48 h after second siRNA transfection, (*, p<0.05, n = 3). The percentage in white indicates the amount of reduction in mRNA content for the targeted gene.

The Ric-8A biosensor results prompted us to focus on a potential role for Ric-8A in cytokinesis. We used Ric-8A siRNAs to reduce Ric8A protein expression. Immunoblot revealed a 70% decrease in protein expression without affecting Gα_i3_ protein expression (Figure 5A) and the Ric-8A immunofluorescent signal at the midbody disappeared upon Ric-8A siRNA treatment (Figure 5B). As we had previously noted extended intercellular bridges following PTX treatment [13], we checked the lengths of intercellular bridges between dividing cells following reduced Ric-8A expression. Compared to control cells the intercellular bridge lengths were increased on average by 3.2 µm (Figure 5B–C). Furthermore, 10% of the dividing Ric-8A siRNA treated cells had intercellular bridges that spanned 18 µm or more (Figure 5C). In the absence of a significant difference in the mitotic index or in the intercellular bridge index (Figure 5D), the increased intercellular bridge length likely results from an abscission defect. To validate this hypothesis, we measured the cell attachment time using a previously described assay [16] (Figure S4A–C in file S1). Using this approach, the mean time that cells remained attached was delayed by 36% in the siRNA Ric-8A treated cells (Figure 5E). To confirm those results, we indirectly measured AuroraB kinase activity, a key player during abscission [25] by evaluating the phosphorylation of one of its substrate (histone H3). Western blot (Figure 5F) or flow cytometry (Figure 5G) techniques showed that Hela cells depleted for Ric-8A protein expression displayed a decrease in Aurora B kinase activity. Since a complete failure of abscission often results in multinucleated cells, we checked whether Ric-8A depletion affected the percentage of multinucleated cells in the culture. We found a significant increase in the number of multinucleated cells, which reached 6.1±1.7% (Figure 5H). This value was comparable to the one achieved by interfering with the expression of RGS14 (Figure 5H), an RGS protein involved in cytokinesis [13]. These results indicate Ric-8A is needed for normal abscission, although not indispensable as abscission occurred in the majority of dividing cells despite the lowered Ric-8A levels.

### Inhibition of Ric-8A or Gα_i_ Activity Decreases the Production of PtdIns(3)P in a Vps34 Activity Dependent Manner

**Figure 6:**
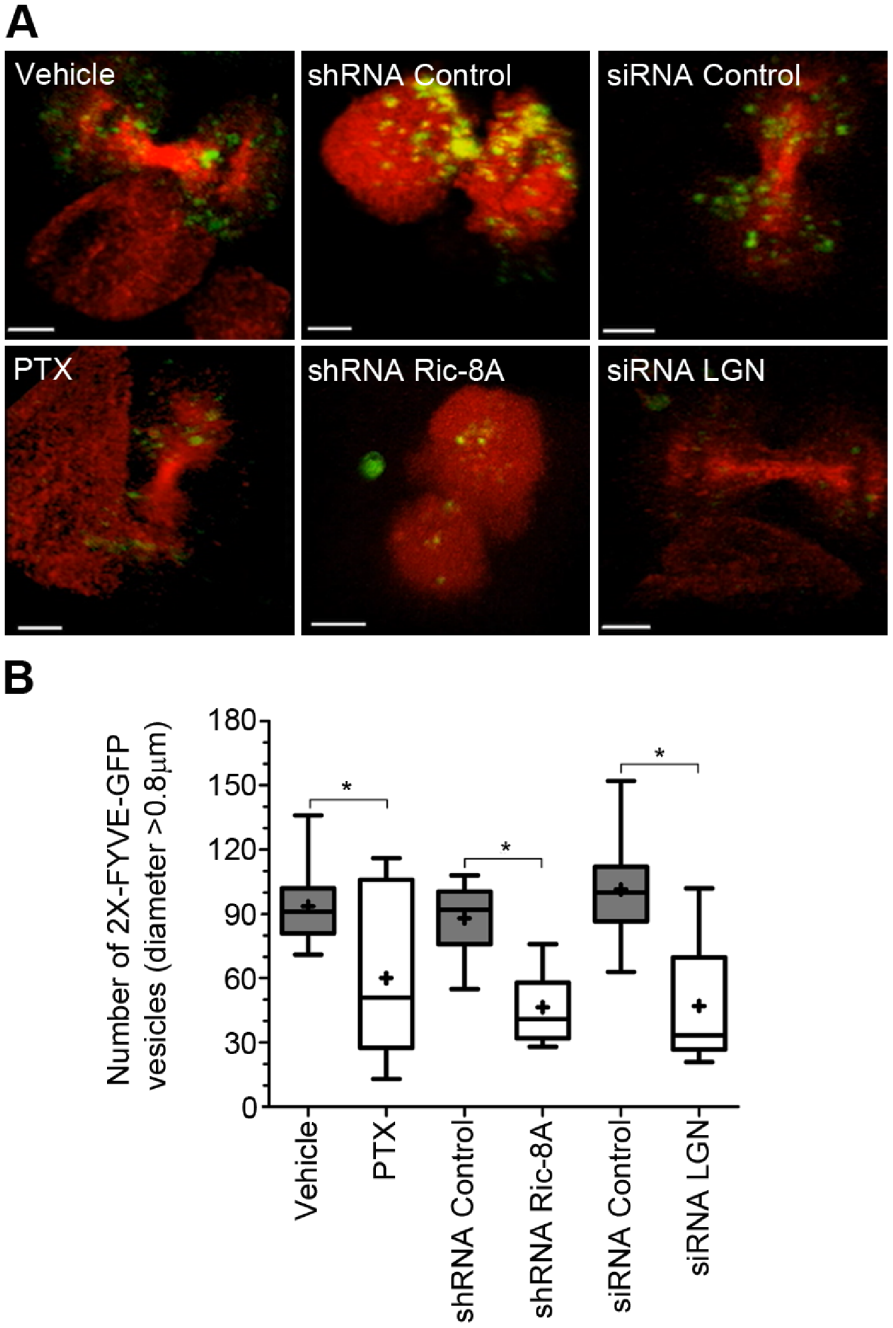
Inhibition of Ric-8A or Gα_i_ activity decreases the recruitment of PtdIns(3)P sensor. (**A**) Snapshots from live cell imaging of GFP-2XFYVE accumulation at the cytokinesis stage. HeLa cells were transiently transfected with GFP-2XFYVE and α-tubulin-RFP (vehicle versus PTX, 2 left panels); GFP-2XFYVE and DsRed-shRNA control or shRNA Ric-8A (middle two panels); or GFP-2XFYVE, α-tubulin-RFP and siRNA control or siRNA LGN (right two panels). Scale bar is 5 µm. Full length movies are available in (movies S1, S2, S3). (**B**) The number of GFP-2XFYVE vesicles was quantified from at least 12 cells in 3 independent experiments and represented on a whisker graph (quartile) where+represents the mean for each condition (*, p<0.05).

Since Ric-8A, Gα_i_ proteins, and RGS14 are located at the midbody and impaired cytokinesis resulted from blocking Gα_i_ nucleotide exchange or inhibiting their expression, we sought to understand the mechanism by which non-canonical G-protein signaling affected cytokinesis. We focused on the activity of Vps34 (vacuolar protein-sorting 34), a class III family member of PI3Ks, which generates phosphatidylinositol-(3)-phosphate (PtdIns(3)P). PtdIns(3)P controls cytokinesis by recruiting a centrosomal protein FYVE-CENT and its binding partner TTC19, which interact with CHMP4B, an ESCRT-III subunit implicated in the abscission step of cytokinesis [26]. To determine if Ric-8A signaling altered the accumulation of PtdIns(3)P in dividing HeLa cells, we expressed GFP-2X-FYVE, a biosensor that binds specifically to PtdIns(3)P [27]. We then treated the cells either with PTX, transfected them with a shRNA targeting Ric-8A, or with siRNAs targeting LGN. In all cases the recruitment of GFP-2X-FYVE to the midbody was reduced compared to the respective control conditions (Figure 6A–B, Movies S1, S2, S3). To confirm these results, we examined the accumulation of PtdIns(3,4)P2, the product of PtdIns(3)P phosphorylation. It can be visualized with AKT^PH^-CFP, a biosensor consisting of the pleckstrin homology (PH) domain of AKT fused to CFP. While AKT^PH^-CFP binds both PtdIns(3,4,5)P3 and PtdIns(3,4)P2 [28], as PtdIns(3,4,5)P3 only accumulates at the polar regions [29] at the onset of cytokinesis, the recruitment of AKT^PH^-CFP to the midbody mostly reflects PtdIns(3,4)P2 levels [30]. We observed that both PTX treatment and a reduction in Ric-8A expression inhibited AKT^PH^-CFP recruitment to the midbody region (Movies S4–S5). These results implicate non-canonical G-protein signaling as an important regulator of PtdIns(3)P production during cytokinesis.

**Figure 7:**
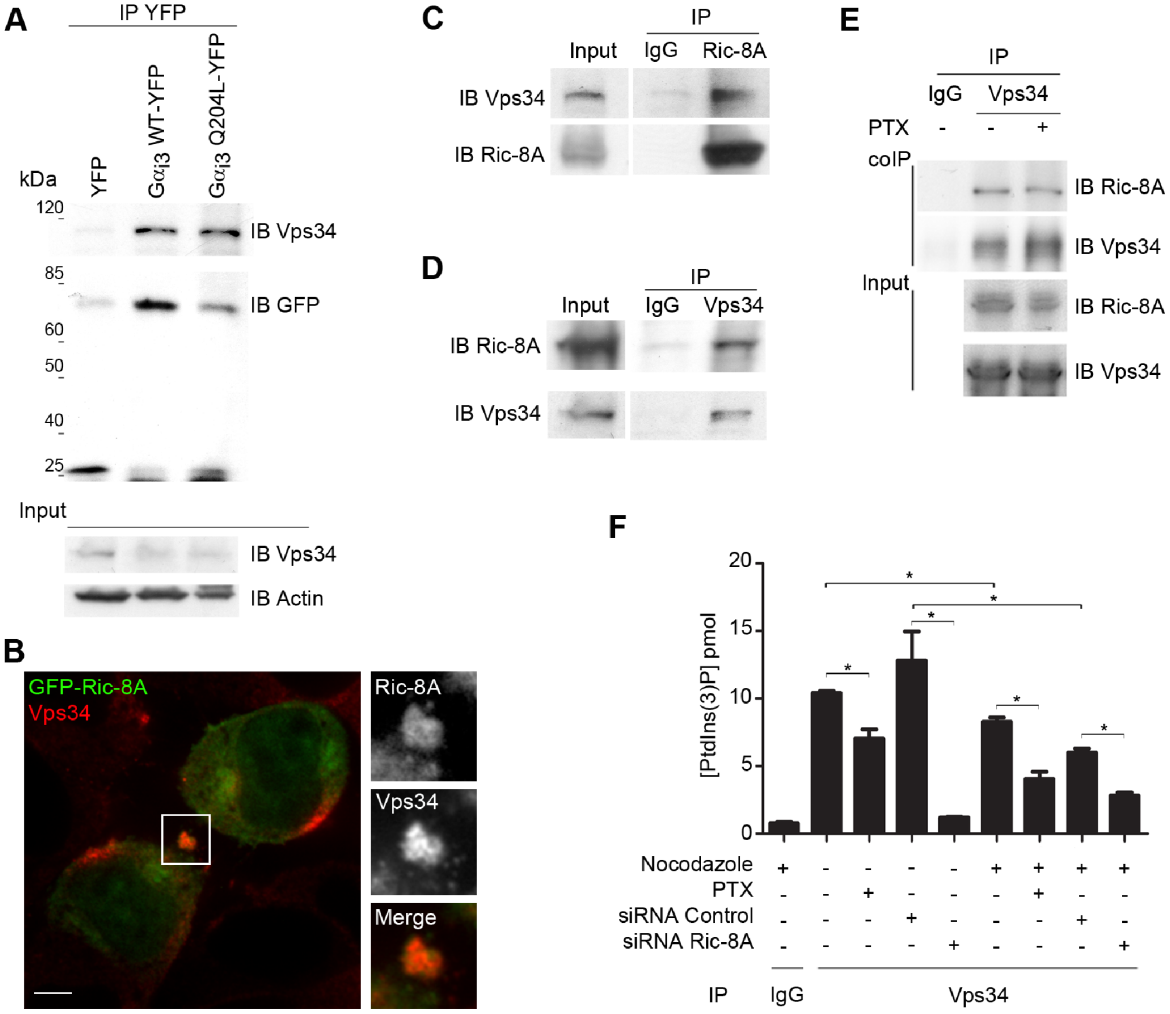
Inhibition of Ric-8A or Gα_i_ activity decreases the production of PtdIns(3)P in a Vps34 activity dependent manner. (**A**) Vps34, GFP and actin immunoblots of cell lysates immunoprecipitate from HeLa cells transiently transfected with vectors expressing YFP, Gα_i3_ wt-YFP, or Gα_i3_ QL-YFP. Experiments were repeated twice with similar results. (**B**) 3D reconstruction of confocal images of HeLa cells expressing GFP-Ric-8A immunostained for endogenous Vps34. Scale bar is 5 µm. The right panels show an electronic magnification of midbody area (white square). (**C–D**) Co-immunoprecipitation of endogenous Vps34 and endogenous Ric-8A in HeLa cells. The experiment was repeated 3 times with similar results. (**E**) Asynchronous HeLa cells lysates treated with PTX (200 ng/mL, 3 h) or with its vehicle were immunoprecipitated using anti-Vps34 antibody and immunoblotted for Ric-8A or Vps34. (**F**) Quantification of PtdIns(3)P production after purification of endogenous Vps34 kinase in asynchronous or G2/M enriched HeLa cells treated with PTX (200 ng/mL, 3 h) or siRNA control versus siRNA Ric-8A. Quantification was performed from 4 independent experiments (*, p<0.05).

A major reason why we focused on PtdIns(3)P is because the Gα_i_ homolog in yeast Gpa1 interacts directly with Vps34 in the endosomes to stimulate PtdIns(3)P production [31]. We confirmed that human Gα_i_ associates with the Vps34 complex as YFP labeled Gα_i3_ and its constitutively active mutant YFP-Gα_i3_ Q204L both efficiently pulled down human Vps34 (Figure 7A). We also documented that Ric-8A and Vps34 colocalized to the midbody during cytokinesis, and that endogenous Ric-8A co-immunoprecipitated with Vps34 (Figure 7B–E) in a PTX insensitive fashion (Figure 7E). To provide direct evidence of the role for Ric-8A and Gα_i_ in the regulation of Vps34 activity, we measured the ability of immunoprecipitated Vps34 to generate PtdIns(3)P under various experimental conditions. We found that PTX treatment or a reduction of Ric-8A levels by RNA interference impaired the production of PtdIns(3)P in asynchronous and G2/M arrested HeLa cells (Figure 7F). These data indicate that Ric-8A, Gα_i_, LGN, and Vps34 localizations at the midbody help to coordinate PtdIns(3)P production and cytokinesis completion.

## Discussion

Our study has shown that in a cancerous cell line, Ric-8A expression level varied during the cell cycle reaching its maximum level at mitosis. A FRET biosensor revealed that Ric-8A undergoes cell cycle dependent changes in its conformation which are most pronounced in the inter-chromosomal area during the later stages of cell division. Confocal microscopy localized Ric-8A along with LGN, Gα_i_ proteins, and RGS14 at the cleavage furrow and midbody. Interference with the expression of Ric-8A, as has been previously shown following knock-down of LGN, RGS14 or Gα_i_ proteins, also result in a cytokinesis defect. Finally, we tied the Ric-8A and Gα_i_ nucleotide exchange to regulation of the phosphatidylinositol 3-kinase activity of Vps34, which is known to participate in cytokinesis.

The importance of mitotic spindle orientation in cancer stem cell biology and the increased frequency of tumor cell multi-nucleation make Ric-8A a candidate for involvement in the transformation of normal cells to cancerous but we didn’t find any trends in Ric-8A protein expression during oncogenesis. Here we found overexpression of Ric-8A proteins in several primary breast cancer tissues, although another study reported a classical double hit genetic inactivation of Ric-8A in a breast cancer cell line and reduced mRNA levels in a subgroup of aggressive TP53 mutant breast cancers [32]. However, we have found that Ric-8A mRNA expression is a poor indicator of Ric-8A protein expression. During the screening of the tumor array, we noted high Ric-8A protein levels in normal testis, a site of rapid cell proliferation. Yet Ric-8A mRNA expression in testis is similar to many non-proliferative tissues. Testing Ric-8A levels as a function of cell cycle position resolved this discrepancy, as we noted a potent upregulation of Ric-8A protein expression during the G2/M phase of the cell cycle. LGN mRNA expression has also been reported to be elevated in human breast cancer tissues, but low in highly proliferative tissues such as testis [24], [33], [34]. In the breast cancer cell line T47D both LGN mRNA and protein expression increased in G2/M cells, suggesting that increased mRNA transcription and/or stability accounted for the protein increases [24], [33]. In contrast to the reported low level of LGN mRNA in testis, querying the Immunological Genome project revealed elevated LGN mRNA expression in rapidly proliferation lymphocytes such as germinal center B cells and double positive thymocyte blasts; and a strong correlation with the expression of other cell cycle dependent genes (http://www.immgen.org/databrowser/index.html). This contrasted with Ric-8A whose mRNA expression varied little between highly proliferative and non-proliferating cells. Together these data indicate that both Ric-8A and LGN protein increase in G2/M phase although via different mechanisms.

We observed a high level of Ric-8A phosphorylation in G2/M phase cells, this observation confirmed previous studies [17] and our site directed mutagenesis correlated phosphoproteomic screen coupled to mass spectrometry identification of serine 501 as a substrate for phosphorylation during mitosis [35]–[37]. Interestingly, we found that this phosphorylation is, at least partially, responsible for Ric-8A ubiquitination that could explain how at a cellular level, HeLa cells can send a molecular signal to reset the amount of Ric-8A protein to start a new G1 phase. The fact that the half-life of Ric-8A is shorter than cell doubling time (t = 14.5±2 h in MEF [3] compare to 20–28 h of MEF doubling time) is compatible with this kind of regulation.

During mitosis, particularly during the cytokinesis stage, we showed that N- and C- termini of Ric-8A moved closer, suggesting a closed state for its conformation. Ric-8A contains multiple helical domains and is predicted to contain 10 armadillo repeats [38]. We tested whether the Ric-8A conformational change depended upon the nucleotide binding status of Gα_i_ or the phosphorylation of Ric-8A at serines 155 or 501 (Figure S5A–B in file S1), however, we found no evidence to support those possibilities. We also examined whether reducing Ric-8A affected Gα_i_ mobility at the midbody area, but we did not observe any change in the kinetic and mobile fraction of Gαi_1–3_ at that site (Figure S6A–C in file S1). Du and Macara used an LGN biosensor to suggest that LGN unhinges during the formation of the LGN/Gα_i_ complex [24]. The Ric-8A conformation change at cytokinesis may also be secondary to an altered interaction with a binding partner such as Vps34, LGN, RGS14, or Gα_i_ [13].

Originally identified in yeast and named for its role in vesicular transport [39], Vps34 binds GTP-bound Gpa1, a yeast Gα_i_ homolog, which promotes increased PtdIns(3)P production [31]. A model in which a Gβ-like protein (Vps15) exists in a preformed complex with the effector enzyme Vps34, and Gα shuttles between these components during a cycle of activation and inactivation has been proposed [31]. Our data indicate that the association of Gα_i_ with a human Vps34 complex is evolutionarily conserved, however the specific interactions are likely different. In human cells, a non-canonical signaling pathway employing Ric-8A, Gα_i_, and LGN function as a molecular switch to regulate the nucleotide status of Gα_i_ and the functional output of Vps34 during cytokinesis. Complexes immunoprecipitated by antibodies directed at either human Vps34 or Vps15 pulled down each other as expected, but also Gα_i_ subunits and Ric-8A (Figure S7 in file S1). There are however several questions which need resolution: How do Gα_i_ and Ric-8A bind to the Vp34/Vps15 complex? Is LGN part of the complex? LGN could serve to anchor Gα_i_ and the LGN/Gα_i_ complex serve as a substrate for Ric-8A triggered nucleotide exchange. Does human Vps15 serve as a Gβ-like protein as it does in yeast [31]? Ric-8A cannot trigger Gα_i_ nucleotide exchange when Gα is bound to Gβγ suggesting that a Vps15/Gα_i_ complex might be insensitive to Ric-8A activity. For several reasons, we do not think that loss of Ric-8A chaperone activity for Gα_i_ subunits [3] could be responsible for the lack of Vps34 activity when Ric-8 expression was inhibited. First, the inhibition of LGN expression resulted in the same phenotype as the use of Ric-8A siRNA or PTX treatment. Second, PTX has been shown to inhibit the GEF activity of Ric-8A without inhibiting its chaperone activity making it unlikely that PTX treatment affected Gα_i_ levels [40]. Third, Gα_i3_ protein expression did not decline when Ric-8A expression was decreased (Figure 5A). Likely the duration of the Ric-8A knock-down was insufficient to cause a decrease in Gα proteins levels. While further studies are needed to resolve the above questions, our data indicate that the Gα_i_ activation and inactivation cycle catalyzed by Ric-8A can regulate cytokinesis.

A further question is how Gα_i_ proteins and Ric-8A are targeted to the midbody area to regulate Vps34 activity? Cytokinesis requires extensive and tightly regulated trafficking of endosomal and exocytic vesicles to the intercellular bridge [41]. This provides a mechanism for the delivery of membrane associated Gα_i_ proteins and other vesicular associate proteins. Gα_i_ could also be transported to the midbody region plasma membrane as a heterotrimeric G protein. Gα proteins typically assemble with Gβγ subunits in the cytosol prior to trafficking to plasma membranes [42]. This would imply that Gβγ subunits should also localize at the midbody region, an assumption which we confirmed as both Gβ_1_ and Gγ_5_ are enriched at the midbody region in dividing HeLa cells (Figure S8 in file S1). This indicates that Gα_i_ is likely, at least partially, associated with Gβγ at this site. Moreover, Eggert and colleagues found involvement of GPCRs in cytokinesis [43] supporting the idea that canonical G-protein signaling also contributes to cytokinesis. Perhaps GPCR triggered Gα_i_ nucleotide exchange release Gα from Gβγ and following GTP hydrolysis, GDP bound Gα_i_ can associate with LGN providing a substrate for Ric-8A. Alternatively, Ric-8A may target a pool of Gα_i_ linked directly to the Vps34 positive vesicles.

While our study has focused on the role of Ric-8A in cytokinesis, the possibility exists that other mammalian Vsp34 complexes associated with autophagy and early endosomes [44] may also be subject to regulation by non-canonical G-protein signaling. Likewise, non-canonical GEFs other than Ric-8A may also be able to trigger Gα_i_ activity to control Vps34 activity. Previous studies report that GIV, a protein which triggers Gα_i3_ GDP/GTP exchange [45], is also involved in the regulation of cell division [46] and autophagy [47]. A role for the Ric-8A/Gα_i_ axis in Vps34 activity during other cellular processes is a plausible hypothesis as the reduction of Ric-8A expression also affected Vps34 activity even in asynchronous cells (Figure 7F).

In conclusion, during mitosis particularly during the cytokinesis stage, we have shown that N- and C-termini of Ric-8A folds into a closed conformation. Despite some effort, we have not identified the cause of this change. We favor the hypothesis that the interaction of Ric-8A with another protein is responsible for the conformational change occurring at cytokinesis. A member of the Vsp34 complex is a reasonable candidate as we found that Ric-8A co-immunoprecipitated with Vps34 and co-localized with it at the midbody.

## Supporting Information

File S1 **Figure S1–S8.**
(DOCX)

Movie S1 **Inhibition of Gαi exchange decreased 2XFYVE-GFP accumulation.** HeLa cells were transfected with 2X-FYVE GFP and mCherry-tubulin and treated with PTX (200 ng/ml for 3 h prior to the experiment, right panel) or its vehicle (left panel) and imaged on a confocal microscope (5 s acquisition for a 22 slices z-stack, 1 image every 3 min). 3D reconstruction was performed using Imaris and movies (2 frames per second) were synchronized using Adobe Premiere. Scale bar is 5 µm.
(MOV)

Movie S2 **Reduced Ric-8A decreased 2XFYVE-GFP accumulation.** HeLa cells were transfected with 2X-FYVE GFP and DsRed- shRNA-Ric8 (right panel) or DsRed-shRNA control (left panel) and imaged on a confocal microscope (5 s acquisition for a 22 slices z-stack, 1 image every 3 min). 3D reconstruction was performed using Imaris and movies (2 frames per second) were synchronized using Adobe Premiere. Scale bar is 5 µm.
(MOV)

Movie S3 **Reduced LGN decreased 2XFYVE-GFP accumulation.** HeLa cells were transfected with 2X-FYVE GFP, mCherry-tubulin and siRNA-LGN (right panel) or siRNA control (left panel) and imaged on a confocal microscope (5 s acquisition for a 22 slices z-stack, 1 image every 3 min). 3D reconstruction was performed using Imaris and movies (2 frames per second) were synchronized using Adobe Premiere. Scale bar is 5 µm.
(MOV)

Movie S4 **Inhibition of Gαi exchange decreased AKT-PH-CFP accumulation.** HeLa cells were transfected with AKT-PH-CFP and mCherry-tubulin and treated with PTX (200 ng/mL 3 h prior experiment, right panel) or its vehicle (left panel) and imaged on a confocal microscope (5 s acquisition for a 22 slices z-stack, 1 image every 3 min). 3D reconstruction was performed using Imaris and movies (2 frames per second) were synchronized using Adobe Premiere. Scale bar is 5 µm.
(MOV)

Movie S5 **Reduced Ric-8A decreased AKT-PH-CFP accumulation.** HeLa cells were transfected with AKT-PH-CFP and DsRed- shRNA-Ric8 (right panel) or DsRed-shRNA control (left panel) and imaged on a confocal microscope (5 s acquisition for a 22 slices z-stack, 1 image every 3 min). 3D reconstruction was performed using Imaris and movies (2 frames per second) were synchronized using Adobe Premiere. Scale bar is 5 µm.
(MOV)

## References

[1] Lambert NA (2008) Dissociation of heterotrimeric g proteins in cells. Sci Signal 1: re5. 18577758 10.1126/scisignal.125re5

[2] Tall GG, Krumins AM, Gilman AG (2003) Mammalian Ric-8A (synembryn) is a heterotrimeric Galpha protein guanine nucleotide exchange factor. J Biol Chem 278: 8356–8362. 12509430 10.1074/jbc.M211862200

[3] Gabay M, Pinter ME, Wright FA, Chan P, Murphy AJ, et al (2011) Ric-8 proteins are molecular chaperones that direct nascent G protein alpha subunit membrane association. Sci Signal 4: ra79. 22114146 10.1126/scisignal.2002223 PMC3870195

[4] Miller KG, Alfonso A, Nguyen M, Crowell JA, Johnson CD, et al (1996) A genetic selection for Caenorhabditis elegans synaptic transmission mutants. Proc Natl Acad Sci U S A 93: 12593–12598. 8901627 10.1073/pnas.93.22.12593 PMC38037

[5] Afshar K, Willard FS, Colombo K, Johnston CA, McCudden CR, et al (2004) RIC-8 is required for GPR-1/2-dependent Galpha function during asymmetric division of C. elegans embryos. Cell 119: 219–230. 15479639 10.1016/j.cell.2004.09.026

[6] Afshar K, Willard FS, Colombo K, Siderovski DP, Gonczy P (2005) Cortical localization of the Galpha protein GPA-16 requires RIC-8 function during C. elegans asymmetric cell division. Development 132: 4449–4459. 16162648 10.1242/dev.02039

[7] Couwenbergs C, Spilker AC, Gotta M (2004) Control of embryonic spindle positioning and Galpha activity by C. elegans RIC-8. Curr Biol 14: 1871–1876. 15498497 10.1016/j.cub.2004.09.059

[8] Woodard GE, Huang NN, Cho H, Miki T, Tall GG, et al (2010) Ric-8A and Gi alpha recruit LGN, NuMA, and dynein to the cell cortex to help orient the mitotic spindle. Mol Cell Biol 30: 3519–3530. 20479129 10.1128/MCB.00394-10 PMC2897540

[9] Siderovski DP, Willard FS (2005) The GAPs, GEFs, and GDIs of heterotrimeric G-protein alpha subunits. Int J Biol Sci 1: 51–66. 15951850 10.7150/ijbs.1.51 PMC1142213

[10] Parmentier ML, Woods D, Greig S, Phan PG, Radovic A, et al (2000) Rapsynoid/partner of inscuteable controls asymmetric division of larval neuroblasts in Drosophila. J Neurosci 20: RC84. 10875939 10.1523/JNEUROSCI.20-14-j0003.2000 PMC6772325

[11] Doherty D, Chudley AE, Coghlan G, Ishak GE, Innes AM, et al (2012) GPSM2 mutations cause the brain malformations and hearing loss in Chudley-McCullough syndrome. Am J Hum Genet 90: 1088–1093. 22578326 10.1016/j.ajhg.2012.04.008 PMC3370271

[12] Blumer JB, Chandler LJ, Lanier SM (2002) Expression analysis and subcellular distribution of the two G-protein regulators AGS3 and LGN indicate distinct functionality. Localization of LGN to the midbody during cytokinesis. J Biol Chem 277: 15897–15903. 11832491 10.1074/jbc.M112185200

[13] Cho H, Kehrl JH (2007) Localization of Gi alpha proteins in the centrosomes and at the midbody: implication for their role in cell division. J Cell Biol 178: 245–255. 17635935 10.1083/jcb.200604114 PMC2064444

[14] Lacroix B, Maddox AS (2012) Cytokinesis, ploidy and aneuploidy. J Pathol 226: 338–351. 21984283 10.1002/path.3013

[15] Krumins AM, Gilman AG (2006) Targeted knockdown of G protein subunits selectively prevents receptor-mediated modulation of effectors and reveals complex changes in non-targeted signaling proteins. J Biol Chem 281: 10250–10262. 16446365 10.1074/jbc.M511551200

[16] Steigemann P, Wurzenberger C, Schmitz MH, Held M, Guizetti J, et al (2009) Aurora B-mediated abscission checkpoint protects against tetraploidization. Cell 136: 473–484. 19203582 10.1016/j.cell.2008.12.020

[17] Yang F, Camp DG 2nd, Gritsenko MA, Luo Q, Kelly RT, et al (2007) Identification of a novel mitotic phosphorylation motif associated with protein localization to the mitotic apparatus. J Cell Sci 120: 4060–4070. 17971412 10.1242/jcs.014795

[18] Moore NL, Narayanan R, Weigel NL (2007) Cyclin dependent kinase 2 and the regulation of human progesterone receptor activity. Steroids 72: 202–209. 17207508 10.1016/j.steroids.2006.11.025 PMC1950255

[19] Knockaert M, Greengard P, Meijer L (2002) Pharmacological inhibitors of cyclin-dependent kinases. Trends Pharmacol Sci 23: 417–425. 12237154 10.1016/s0165-6147(02)02071-0

[20] Koepp DM, Schaefer LK, Ye X, Keyomarsi K, Chu C, et al (2001) Phosphorylation-dependent ubiquitination of cyclin E by the SCFFbw7 ubiquitin ligase. Science 294: 173–177. 11533444 10.1126/science.1065203

[21] Lin DI, Barbash O, Kumar KG, Weber JD, Harper JW, et al (2006) Phosphorylation-dependent ubiquitination of cyclin D1 by the SCF(FBX4-alphaB crystallin) complex. Mol Cell 24: 355–366. 17081987 10.1016/j.molcel.2006.09.007 PMC1702390

[22] Dalrymple MB, Jaeger WC, Eidne KA, Pfleger KD (2011) Temporal profiling of orexin receptor-arrestin-ubiquitin complexes reveals differences between receptor subtypes. J Biol Chem 286: 16726–16733. 21378163 10.1074/jbc.M111.223537 PMC3089514

[23] Perroy J, Pontier S, Charest PG, Aubry M, Bouvier M (2004) Real-time monitoring of ubiquitination in living cells by BRET. Nat Methods 1: 203–208. 15782195 10.1038/nmeth722

[24] Du Q, Macara IG (2004) Mammalian Pins is a conformational switch that links NuMA to heterotrimeric G proteins. Cell 119: 503–516. 15537540 10.1016/j.cell.2004.10.028

[25] Carmena M (2012) Abscission checkpoint control: stuck in the middle with Aurora B. Open Biol. 2: 120095. 10.1098/rsob.120095 PMC3411112 22870391

[26] Sagona AP, Nezis IP, Pedersen NM, Liestol K, Poulton J, et al (2010) PtdIns(3)P controls cytokinesis through KIF13A-mediated recruitment of FYVE-CENT to the midbody. Nat Cell Biol 12: 362–371. 20208530 10.1038/ncb2036

[27] Gillooly DJ, Morrow IC, Lindsay M, Gould R, Bryant NJ, et al (2000) Localization of phosphatidylinositol 3-phosphate in yeast and mammalian cells. EMBO J 19: 4577–4588. 10970851 10.1093/emboj/19.17.4577 PMC302054

[28] Kavran JM, Klein DE, Lee A, Falasca M, Isakoff SJ, et al (1998) Specificity and promiscuity in phosphoinositide binding by pleckstrin homology domains. J Biol Chem 273: 30497–30508. 9804818 10.1074/jbc.273.46.30497

[29] Janetopoulos C, Borleis J, Vazquez F, Iijima M, Devreotes P (2005) Temporal and spatial regulation of phosphoinositide signaling mediates cytokinesis. Dev Cell 8: 467–477. 15809030 10.1016/j.devcel.2005.02.010

[30] Toyoshima F, Matsumura S, Morimoto H, Mitsushima M, Nishida E (2007) PtdIns(3,4,5)P3 regulates spindle orientation in adherent cells. Dev Cell 13: 796–811. 18061563 10.1016/j.devcel.2007.10.014

[31] Slessareva JE, Routt SM, Temple B, Bankaitis VA, Dohlman HG (2006) Activation of the phosphatidylinositol 3-kinase Vps34 by a G protein alpha subunit at the endosome. Cell 126: 191–203. 16839886 10.1016/j.cell.2006.04.045

[32] Muggerud AA, Edgren H, Wolf M, Kleivi K, Dejeux E, et al (2009) Data integration from two microarray platforms identifies bi-allelic genetic inactivation of RIC8A in a breast cancer cell line. BMC Med Genomics 2: 26. 19432969 10.1186/1755-8794-2-26 PMC2685142

[33] Fukukawa C, Ueda K, Nishidate T, Katagiri T, Nakamura Y (2010) Critical roles of LGN/GPSM2 phosphorylation by PBK/TOPK in cell division of breast cancer cells. Genes Chromosomes Cancer 49: 861–872. 20589935 10.1002/gcc.20795

[34] Kao J, Salari K, Bocanegra M, Choi YL, Girard L, et al (2009) Molecular profiling of breast cancer cell lines defines relevant tumor models and provides a resource for cancer gene discovery. PLoS One 4: e6146. 19582160 10.1371/journal.pone.0006146 PMC2702084

[35] Alexander J, Lim D, Joughin BA, Hegemann B, Hutchins JR, et al (2011) Spatial exclusivity combined with positive and negative selection of phosphorylation motifs is the basis for context-dependent mitotic signaling. Sci Signal 4: ra42. 21712545 10.1126/scisignal.2001796 PMC3939359

[36] Mayya V, Lundgren DH, Hwang SI, Rezaul K, Wu L, et al (2009) Quantitative phosphoproteomic analysis of T cell receptor signaling reveals system-wide modulation of protein-protein interactions. Sci Signal 2: ra46. 19690332 10.1126/scisignal.2000007

[37] Huttlin EL, Jedrychowski MP, Elias JE, Goswami T, Rad R, et al (2010) A tissue-specific atlas of mouse protein phosphorylation and expression. Cell 143: 1174–1189. 21183079 10.1016/j.cell.2010.12.001 PMC3035969

[38] Figueroa M, Hinrichs MV, Bunster M, Babbitt P, Martinez-Oyanedel J, et al (2009) Biophysical studies support a predicted superhelical structure with armadillo repeats for Ric-8. Protein Sci 18: 1139–1145. 19472323 10.1002/pro.124 PMC2774424

[39] Wurmser AE, Gary JD, Emr SD (1999) Phosphoinositide 3-kinases and their FYVE domain-containing effectors as regulators of vacuolar/lysosomal membrane trafficking pathways. J Biol Chem 274: 9129–9132. 10092582 10.1074/jbc.274.14.9129

[40] Oner SS, Maher E, Gabay M, Tall GG, Blumer JB, et al.. (2012) Regulation of the GPR-Galphai Signaling Complex by non-receptor Guanine Nucleotide Exchange Factors. J Biol Chem. 10.1074/jbc.M112.418467 PMC3561525 23212907

[41] Montagnac G, Echard A, Chavrier P (2008) Endocytic traffic in animal cell cytokinesis. Curr Opin Cell Biol 20: 454–461. 18472411 10.1016/j.ceb.2008.03.011

[42] Neer EJ (1995) Heterotrimeric G proteins: organizers of transmembrane signals. Cell 80: 249–257. 7834744 10.1016/0092-8674(95)90407-7

[43] Zhang X, Bedigian AV, Wang W, Eggert US (2012) G protein-coupled receptors participate in cytokinesis. Cytoskeleton (Hoboken) 69: 810–818. 22888021 10.1002/cm.21055 PMC4137143

[44] Funderburk SF, Wang QJ, Yue Z (2010) The Beclin 1-VPS34 complex–at the crossroads of autophagy and beyond. Trends Cell Biol 20: 355–362. 20356743 10.1016/j.tcb.2010.03.002 PMC3781210

[45] Garcia-Marcos M, Ghosh P, Farquhar MG (2009) GIV is a nonreceptor GEF for G alpha i with a unique motif that regulates Akt signaling. Proc Natl Acad Sci U S A 106: 3178–3183. 19211784 10.1073/pnas.0900294106 PMC2651282

[46] Mao JZ, Jiang P, Cui SP, Ren YL, Zhao J, et al.. (2012) Girdin locates in centrosome and midbody and plays an important role in cell division. Cancer Sci. 10.1111/j.1349-7006.2012.02378.x PMC7659294 22755556

[47] Garcia-Marcos M, Ear J, Farquhar MG, Ghosh P (2011) A GDI (AGS3) and a GEF (GIV) regulate autophagy by balancing G protein activity and growth factor signals. Mol Biol Cell 22: 673–686. 21209316 10.1091/mbc.E10-08-0738 PMC3046063

