# Supplementary material for: Resistance to Inhibitors of Cholinesterase (Ric)-8A and Gαi Contribute to Cytokinesis Abscission by Controlling Vacuolar Protein-Sorting (Vps)34 Activity
Source: PLoS One. 2014 Jan 21;9(1):e86680. doi: 10.1371/journal.pone.0086680 (PMC3897744; doi:10.1371/journal.pone.0086680)
Supplement: File S1 — Figure S1–S8. (DOCX) [file pone.0086680.s001.docx]

**SUPPLEMENTARY INFORMATION**

**Resistance to inhibitors of cholinesterase (Ric)-8A and Gα_i_ contribute to cytokinesis abscission by controlling vacuolar protein-sorting (Vps)34 activity**

**Cedric BOULARAN ^1^, Olena KAMENYEVA^1^, Hyeseon CHO^1^ and John H. KEHRL ^1^**

**^1^**B-cell Molecular Immunology Section, Laboratory of Immunoregulation, National Institutes of Allergy and Infectious Diseases, National Institutes of Health, Bethesda, Maryland, United States of America

To whom correspondence should be addressed: John H. KEHRL, Laboratory of Immunoregulation, National Institute of Allergy and Infectious Diseases, National Institutes of Health, Bldg. 10, Room 11B08, 10 Center Dr. MSC 1876, Bethesda, Maryland 20892, United States of America; Fax: 301-402-0070. E-mail: [jkehrl@niaid.nih.gov](mailto:jkehrl@niaid.nih.gov)

**CONTENTS**

FIGURES S1-S8

SUPPORTING MOVIES LEGENDS S1-S5

**SUPPLEMENTARY FIGURES**

**

**

**Figure S1 Ric-8A phosphorylation occurs on a serine**

Representative Ric-8A, phospho-serine and actin immunoblots of anti- phospho-p190 or anti Ric-8A antibodies immunoprecipitates prepared from asynchronous or nocodazole G2/M arrested HeLa cells. Serine phosphatases were inhibited by okadaic acid (10 μM) only during immunoprecipitation and immunoprecipitation was performed at room temperature to allow full activity of endogenous serine phosphatases.

**
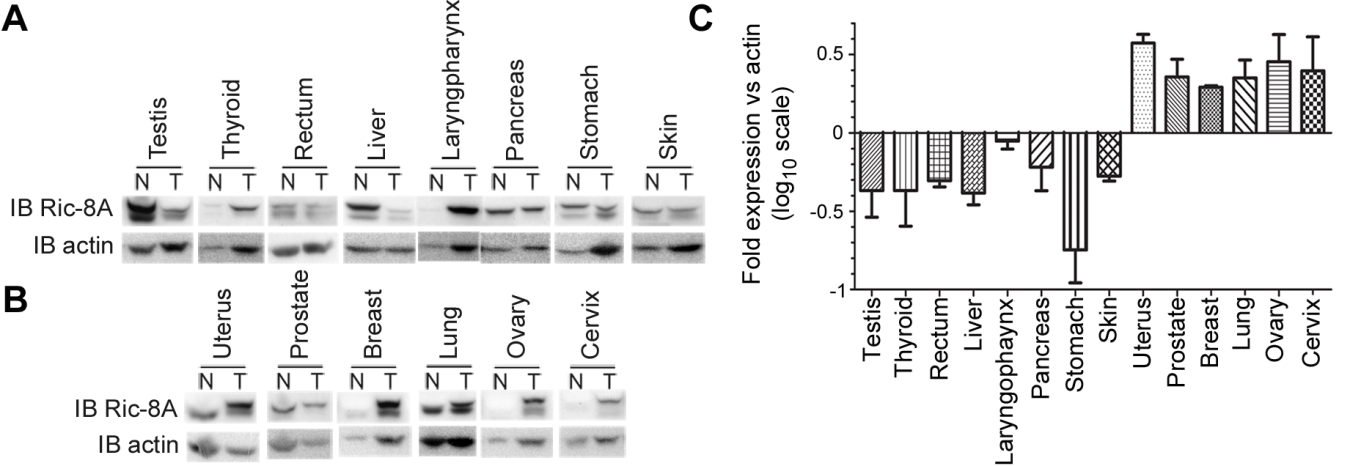
**

**Figure S2 Ric-8A protein expression is altered in primary tumors.**

(**A-B**) Ric-8A and actin immunoblots of tumor arrays spotted with tissue lysates from patient biopsies of cancerous or non-tumor tissues (age- and sex- matched). (**C**) Endogenous Ric-8A expression was normalized to actin and expressed as a fold expression in cancer sample vs. normal one from 2 independent experiments using 2 batches of membranes (GBiosciences).

**
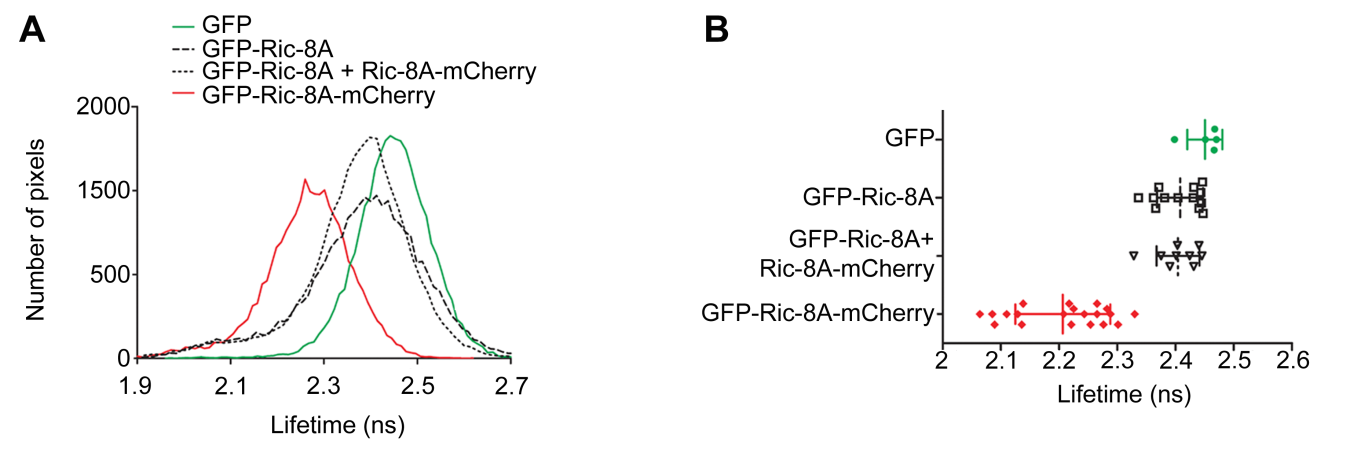
**

**Figure S3 Ric-8A conformational change is intra-molecular mediated**

HeLa cells were transiently transfected with GFP, GFP-Ric-8A, GFP-Ric-8A and Ric-8A-mCherry, or GFP-Ric-8A-mCherry for 24 h. FRET by FLIM measurements were done using live cells. Lifetime distribution in a representative cell (**A**) and lifetime average across 10-20 cells (**B**) show that FRET measurement from GFP-Ric-8A-mCherry is intra-molecular not inter-molecular.

**
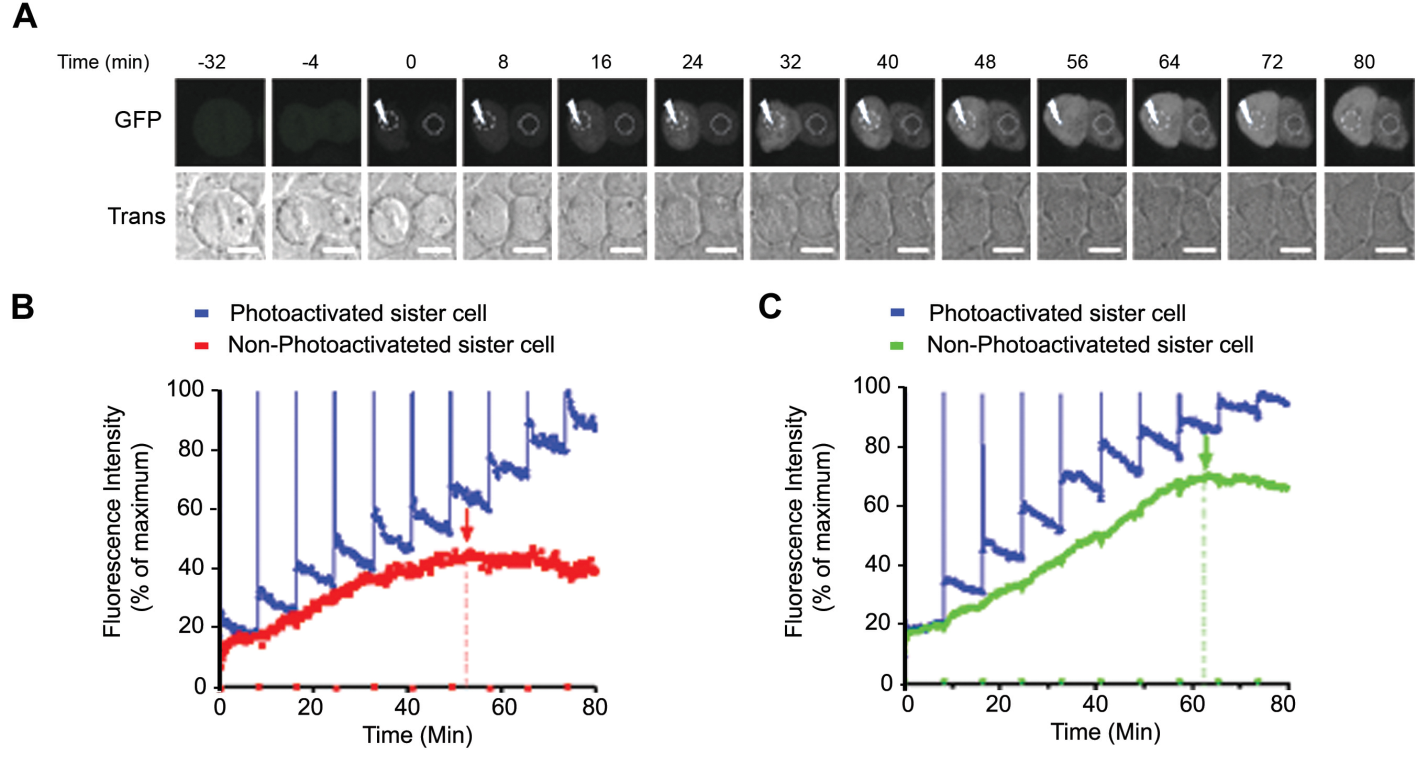
**

**Figure S4 Ric-8A inhibition delays the cytokinesis abscission time**.

(**A**) Focusing on metaphase cells, PA-GFP was induced during telophase by a single laser pulse at 405 nm using 100% of the laser power in an approximately 5 μm^2^ area of 1 sister cell (lightning in dashed circle). After activation, the GFP signal was collected on single section at 15 s intervals during 8 min. This cycle of photoactivation and recording were repeated 10 times to cover the whole length of mitosis. Quantification of the mean GFP signals in the region of interest (dashed circle) in the photoactivated sister cell and in a region of the same size in the non-photoactivated sister cells is plotted. Abscission was determined by the stop in fluorescence increase in the non-photoactivated sister cell as indicated by the arrows for siRNA control treated cell (**B**) or siRNA Ric-8A treated cell (**C**).

**
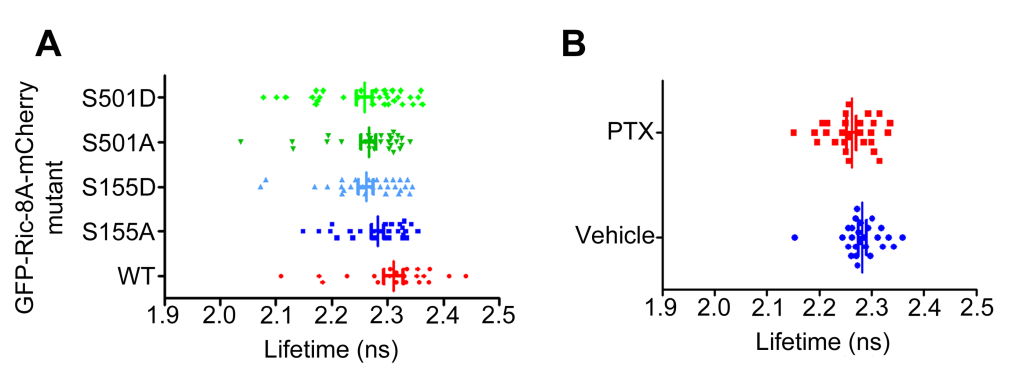
**

**Figure S5 Ric-8A conformational change is S155, S501-phosphorylation, Gα_i_ activity independent.**

**(A**) Lifetime average across 10-20 cells transiently transfected with the indicated mutant show that intra-molecular FRET is independent of phosphorylation events on S155 or S501 **(B**) Lifetime average across 10-20 cells transiently transfected with GFP-Ric-8A-mCherry and treated with PTX (200 ng/ml, 3 h) or with its vehicle shows that FRET measurement is Gα_i_ activity independent.


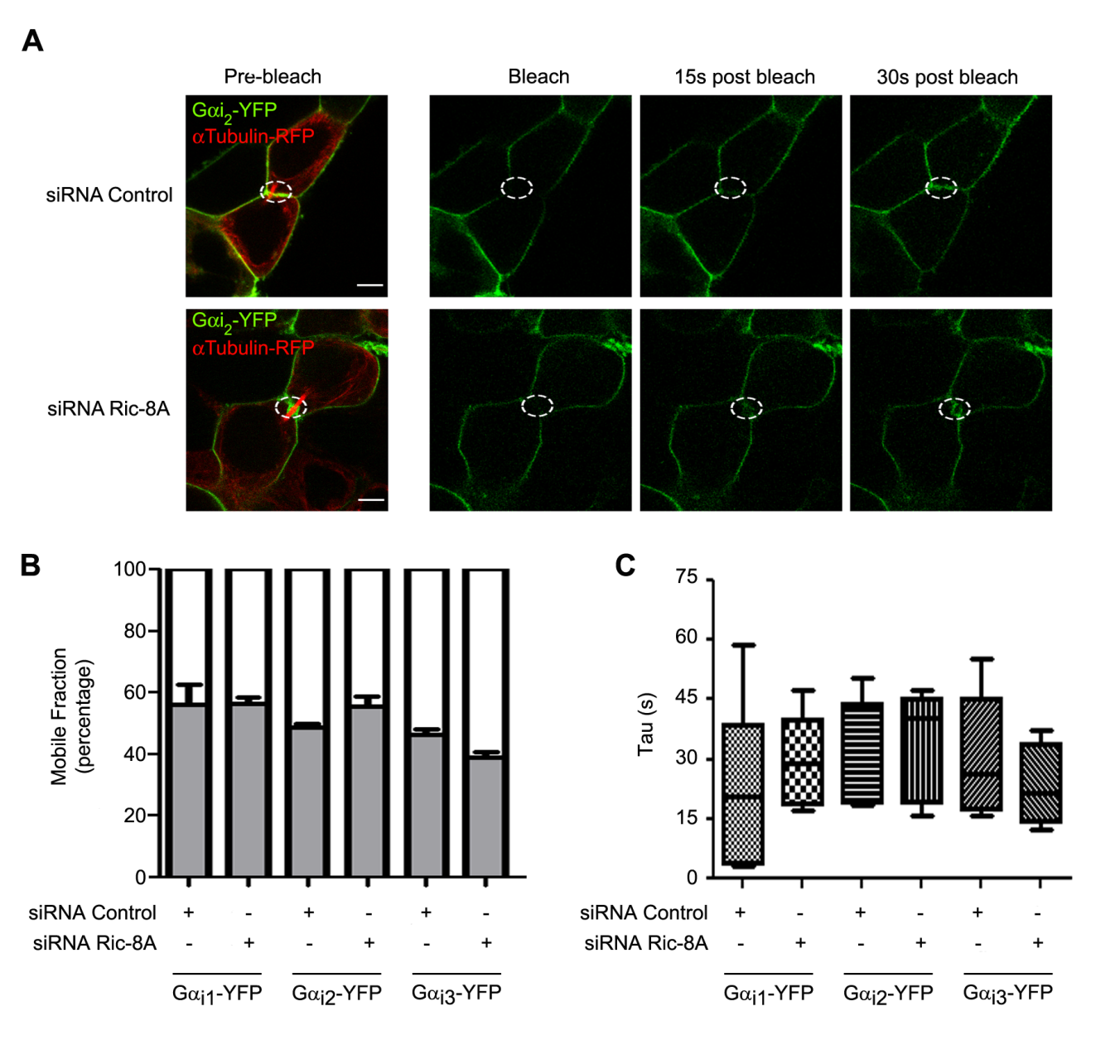


**Figure S6 Gα_i_ mobility and localization at the midbody is Ric-8A independent.**

(**A**) HeLa cells were transiently transfected with YFP tagged Gα_i2_ and siRNA control or siRNA targeting Ric-8A for 48 h. A representative FRAP experiment show the fluorescence recovery 15 and 30 s after bleaching the midbody area, Scale bar is 5 μm (**B**) Quantification of mobile fraction (grey histogram) show that Ric-8A inhibition doesn’t affect the percentage of the different Gα_i_ isoforms mobile fraction. (**C**) Quantification of the fluorescence recovering time (whisker bar representation of 15 cells acquired during 3 independent experiments) show that Gα_i_ mobility is Ric-8A independent

**
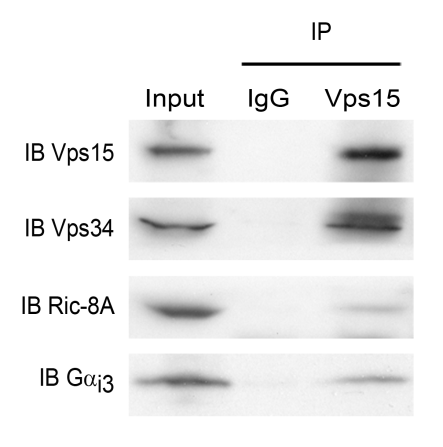
**

**Figure S7 Vps34-Ric-8A complex can include Vps15 and Gα_i3_.**

Co-immunoprecipitation of endogenous Vps15, endogenous Vps34, endogenous Ric-8A and endogenous Gα_i3_ in HeLa cells. The experiment was repeated 2 times with similar results.

**
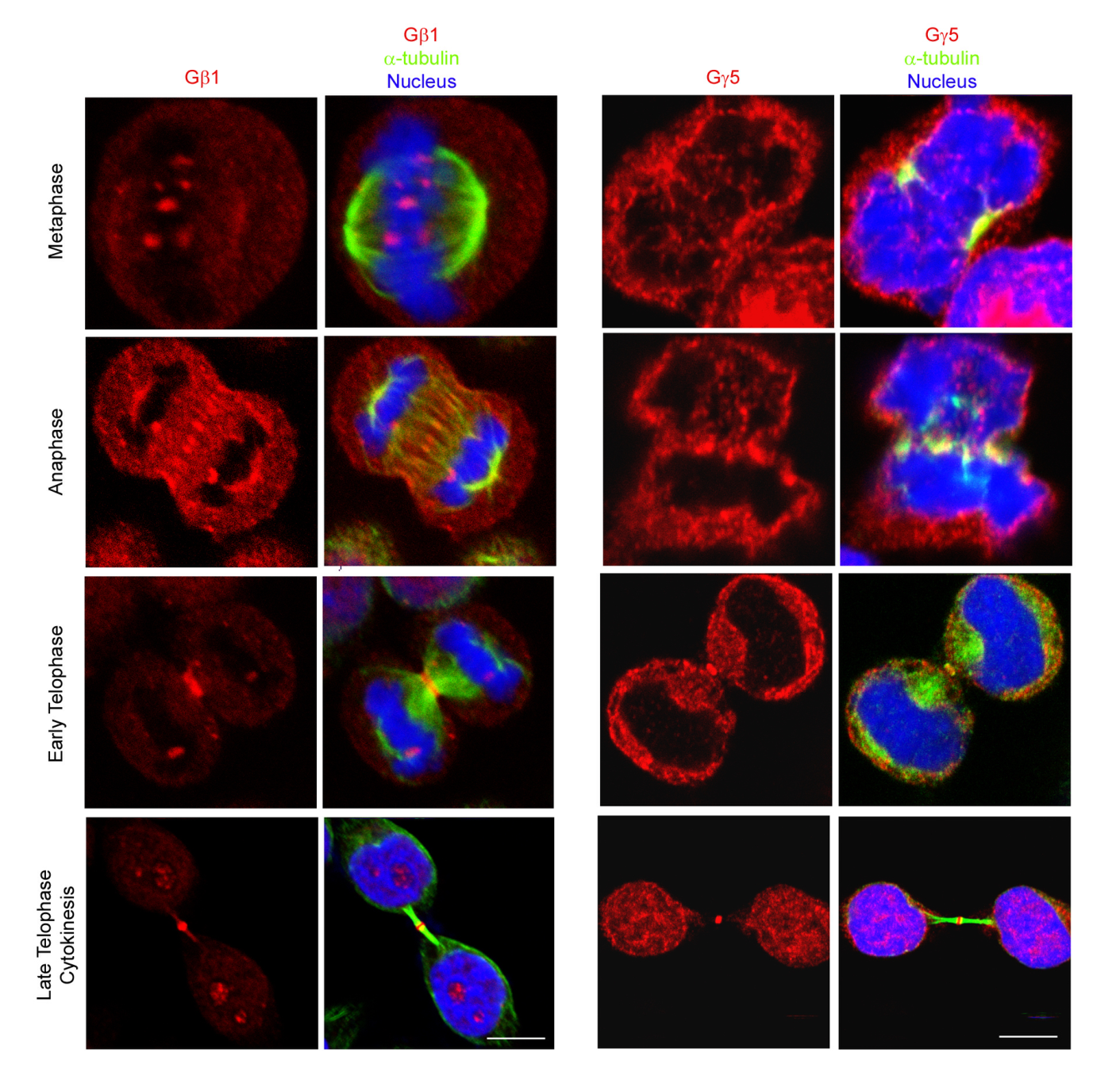
**

**Figure S8 G_β1_ and G_γ5_ are localized to the midbody.**

Asynchronous HeLa cells were immunostained for G_β1_ and G_γ5_ after Methanol/acetone fixation-permeabilization. Quadrants show the endogenous localization of the proteins through the cell cycle from metaphase to telophase identified based on the alpha tubulin and nuclei stainings. Scale bar is 10 μm. The experiment was repeated 2 times with similar results.

**SUPPORTING MOVIES LEGENDS**

**Movie S1** Inhibition of Gα_i_ exchange decreased 2XFYVE-GFP accumulation. HeLa cells were transfected with 2X-FYVE GFP and mCherry-tubulin and treated with PTX (200 ng/ml for 3 h prior to the experiment, right panel) or its vehicle (left panel) and imaged on a confocal microscope (5 s acquisition for a 22 slices z-stack, 1 image every 3 min). 3D reconstruction was performed using Imaris and movies (2 frames per second) were synchronized using Adobe Premiere. Scale bar is 5 μm.

**Movie S2** Reduced Ric-8A decreased 2XFYVE-GFP accumulation. HeLa cells were transfected with 2X-FYVE GFP and DsRed- shRNA-Ric8 (right panel) or DsRed-shRNA control (left panel) and imaged on a confocal microscope (5 s acquisition for a 22 slices z-stack, 1 image every 3 min). 3D reconstruction was performed using Imaris and movies (2 frames per second) were synchronized using Adobe Premiere. Scale bar is 5 μm.

**Movie S3** Reduced LGN decreased 2XFYVE-GFP accumulation. HeLa cells were transfected with 2X-FYVE GFP, mCherry-tubulin and siRNA-LGN (right panel) or siRNA control (left panel) and imaged on a confocal microscope (5 s acquisition for a 22 slices z-stack, 1 image every 3 min). 3D reconstruction was performed using Imaris and movies (2 frames per second) were synchronized using Adobe Premiere. Scale bar is 5 μm.

**Movie S4** Inhibition of Gα_i_ exchange decreased AKT-PH-CFP accumulation. HeLa cells were transfected with AKT-PH-CFP and mCherry-tubulin and treated with PTX (200 ng/mL 3 h prior experiment, right panel) or its vehicle (left panel) and imaged on a confocal microscope (5 s acquisition for a 22 slices z-stack, 1 image every 3 min). 3D reconstruction was performed using Imaris and movies (2 frames per second) were synchronized using Adobe Premiere. Scale bar is 5 μm.

**Movie S5** Reduced Ric-8A decreased AKT-PH-CFP accumulation. HeLa cells were transfected with AKT-PH-CFP and DsRed- shRNA-Ric8 (right panel) or DsRed-shRNA control (left panel) and imaged on a confocal microscope (5 s acquisition for a 22 slices z-stack, 1 image every 3 min). 3D reconstruction was performed using Imaris and movies (2 frames per second) were synchronized using Adobe Premiere. Scale bar is 5 μm.
